# Effectiveness of interventions for anxiety and depression in children and young people: an umbrella review of meta-analyses

**DOI:** 10.1007/s00787-026-03064-w

**Published:** 2026-05-26

**Authors:** Isabella Morse, Julia Mannes, Dihini Pilimatalawwe, Samantha Phippard, Fabienne dos Santos Sousa, Rani Samuel, Mohan Ganesalingam, Emma Rachel Rengasamy, Carmen Pinto, Matt Woolgar, Robbie Duschinsky, Barry Coughlan

**Affiliations:** 1 Child Health and Development Group, Department of Public Health and Primary Care, University of Cambridge, Cambridge, UK; 2 Lewisham CAMHS, South London and Maudsley NHS Foundation Trust, London, UK; 3 Norfolk and Suffolk NHS Foundation Trust, Norwich, UK; 4 South London and Maudsley NHS Foundations Trust, London, UK; 5 Institute of Psychiatry, King’s College London, Psychology & Neuroscience (IoPPN), London, UK; 6 Department of Social Sciences, National College of Ireland, Dublin, Ireland

**Keywords:** Anxiety, Depression, Children and young people, Interventions, Umbrella review

## Abstract

Anxiety and depression are the most common mental health conditions for children and young people (CYP), yet treatment recommendations vary. Through this umbrella review (meta-analysis of meta-analyses), we aimed to identify the most effective interventions for CYP with anxiety and depression. We searched five electronic databases (PubMed, Medline, PsycINFO, Web of Science, ASSIA) from 1 October 2017 to 1 October 2022, with an updated search in PubMed from October 2022 to February 2026. Eligible meta-analyses reported on interventions for CYP (≤ 18 years) with anxiety or depression and/or associated symptoms. We identified 57 meta-analyses (569 effect sizes); of these, 43 (391 effects) were included in statistical analyses. For anxiety, psychosocial (d = -0.51, 95% CI [-0.56, -0.46], p < 0.001) and pharmacological (d = -0.67, 95% CI [-0.87, -0.46], p < 0.001) interventions appeared equally effective (t(5.50) = 0.64, p = 0.546). For depression, physical (d = -0.49, CI [-0.57, -0.40], p < 0.001) interventions appeared most effective, followed by psychosocial (d = -0.35, CI [-0.39, -0.32], p < 0.001), then pharmacological (d = -0.17, CI [-0.20, -0.15], p < 0.001), (F(2,151) = 11.44, p < 0.001). Psychosocial interventions were also associated with better outcomes for anxiety than for depression (MD = -0.14, CI [-0.21, -0.06], t(314.30) = -3.45, p < 0.001). As physical interventions and interpersonal therapy were the specific therapies associated with the greatest improvements in CYP’s depression, policymakers and practitioners should monitor emerging evidence in these areas when shaping treatment recommendations, and researchers should consider exploring their effectiveness for the treatment of anxiety. In general, future research should focus on understanding the effectiveness of the studied interventions in real clinical practice settings.

**Supplementary Information:**

The online version contains supplementary material available at https://doi.org/10.1007/s00787-026-03064-w.

## Introduction

Anxiety and depression are the most common mental health conditions among children and young people (CYP) [1]. A recent meta-analysis of over 80,000 CYP found clinically significant anxiety in one in five and depression in one in four [2], with similar rates reported elsewhere [3–5]. Untreated anxiety and depression in CYP are associated with negative long-term outcomes [6–8]. Cognitive behavioral therapy (CBT) and second-generation antidepressants (including selective serotonin reuptake inhibitors (SSRIs)) are among the most common interventions for both [4, 9–12]. Interventions can be broadly categorized as psychosocial or pharmacological. Additionally, in the last two decades, physical interventions (e.g., exercise therapies) have been increasingly explored for depression [13–16].

There is discussion among policymakers, practitioners, and researchers surrounding the relative effectiveness of treatments [17]. Meta-analyses are common, but they usually focus on specific treatments and can present competing findings, sustaining debate. For CYP with anxiety disorders, one meta-analysis found CBT more effective than second-generation antidepressants [18], while an alternative network meta-analysis found only second-generation antidepressants, not CBT, were more effective than placebo [19]. Meanwhile, governments and nonprofits promote CBT as a first-line treatment for anxious CYP, but maintain ambiguity about second-generation antidepressants, particularly in “severe” cases [20, 21]. Non-antidepressant pharmacological interventions, such as benzodiazepines and antipsychotics, are not recommended for routine treatment of anxiety disorders, especially in CYP, due to potentially dangerous side effects [11].

Similarly, for CYP with depression, several studies and meta-analyses present contradictory findings, suggesting that either CBT [22, 23] or second-generation antidepressants [24–26] are the more effective long-term treatment. Public health guidance is also mixed: most British bodies suggest that second-generation antidepressants should never be a standalone first-line treatment for CYP with depression, given the potential risks and side effects [12, 26], while some American bodies endorse it as a viable first-line option [27]. As for anxiety, when second-generation antidepressants are recommended for depression, it is primarily for older CYP with severe and enduring symptoms and is often in combination with psychosocial interventions [28]. Physical interventions have been increasingly studied for CYP with depression, primarily at the mild to moderate level, and appear effective [29] but are rarely a recommended treatment option.

Still other meta-analyses have found no significant difference in the efficacy of psychosocial versus pharmacological interventions for anxiety or depression [30, 31].

Despite the debates surrounding the treatment of anxiety and depression in CYP, researchers, practitioners, and policymakers agree on the importance of evidence-based interventions [11, 12, 32, 33]. This umbrella review aims to provide a comprehensive synthesis of research on interventions for CYP with anxiety and depression to help inform policy and practice, by answering the following questions:

1. What interventions are provided for CYP with anxiety and/or depression?
2. How effective are these interventions in treating CYP’s symptoms of anxiety and depression and improving remission rates?

## Methods

### Search strategy

**Fig. 1:**
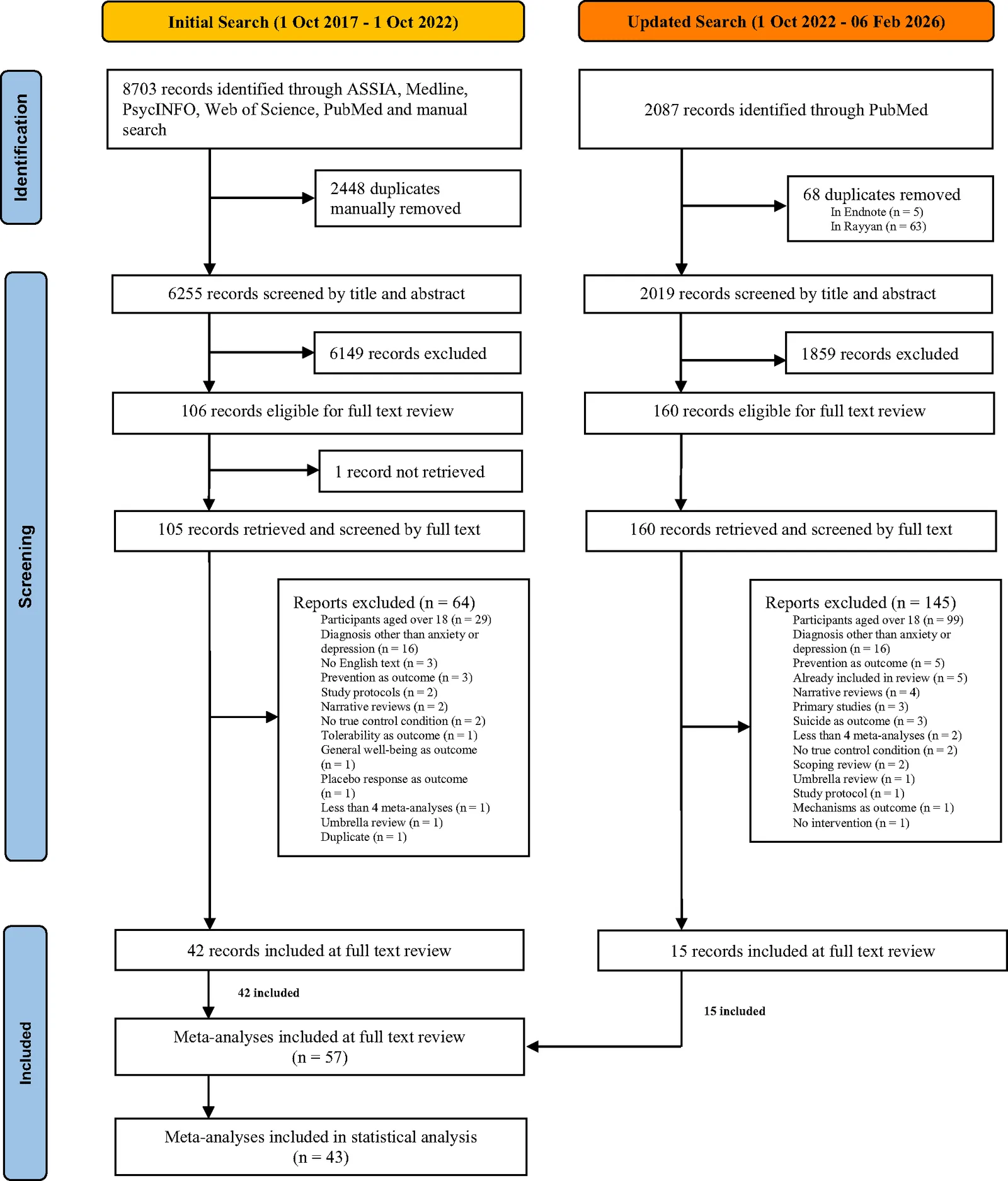
Study selection: PRISMA flow diagram. Source: [35] https://doi.org/10.1136/bmj.n71. Note: ASSIA = Applied Social Sciences Index & Abstracts

This umbrella review (a meta-analysis of meta-analyses) followed best practice guidance [34]. A search of five electronic databases (PubMed, Medline, PsycINFO, Web of Science, ASSIA) was conducted on 1 October 2022. The search was limited to five years (1 October 2017 to 1 October 2022) after a preliminary ten-year search indicated potential for significant overlap in studies between older and newer meta-analyses (See Supplementary S1-S2). The search was rerun in PubMed on 6 February 2026 for publications from 1 October 2022 to 6 February 2026 to incorporate the most updated findings. The PubMed database was selected for the updated search due to feasibility constraints, as it indexes the majority of journals captured by the original databases (MEDLINE, PsycINFO, Web of Science, and ASSIA). Study selection followed PRISMA guidelines [35] (see Fig. 1).

The initial search produced 8703 results; after manual removal of duplicates, 6255 records remained and were brought to title and abstract screening. Of these, 106 were brought to full text review, and 42 were deemed to meet inclusion criteria.

The updated 2026 search produced 2087 records, and 2019 remained after duplicates were identified via Endnote and Rayaan. Of these, 160 were brought to full text review, and 15 new articles were deemed to meet inclusion criteria.

### Selection criteria and data selection

Meta-analytic and systematic reviews from any country were accepted provided they included CYP aged 18 and younger with anxiety or depression symptoms/diagnoses. Eligible mental health interventions were pharmacological, psychosocial (including family and group therapies), and physical. Only English-language publications were included due to lack of translation resources and to ensure accessibility for all screeners (See Supplementary S3 for full criteria).

For the initial search, a random 46% of publications were double-screened at the title and abstract phase by the first author and one of two additional reviewers. The prevalence-adjusted and bias-adjusted kappa (PABAK) was calculated, finding excellent agreement of 0.98 [36]. Given high agreement, the remaining articles were screened by the first author only. Title and abstract screening for the updated search was also completed by a single author. 106 publications were identified at this stage and brought to full text review (See Fig. 1).

All publications were screened by at least two researchers at full text review. When two reviewers disagreed on an inclusion decision, a third researcher reviewed the article, and their decision resolved the disagreement. In the initial search, 42 articles met inclusion criteria for the umbrella review and 64 failed to meet these criteria. In the updated search, 15 articles met inclusion criteria and 140 did not (See Fig. 1).

### Data analysis and quality

Characteristics and outcomes from included articles were extracted during this review stage, with all data double-coded and collated, and/or checked by the first author against the original text. Disagreements were resolved by discussion and reference to text. Data extraction of the 42 articles from the initial search resulted in 520 effect sizes, and extraction of the 15 articles from the updated search produced an additional 49 effects (See Supplementary S4 for data categories extracted). Meta-analyses were assessed for substantial overlap in primary studies, with a threshold of 75% established a priori. Meta-analyses in the same analytical categories were assessed, and where over 75% of primary studies from one meta-analysis were contained within another meta-analysis, the meta-analysis with fewer primary studies was excluded. A total of 12 meta-analyses with high overlap were identified in this way and excluded from at least one level of analysis.

As most studies reported outcomes in Cohen’s *d*, all other effect sizes were transformed to Cohen’s *d* to facilitate comparison. Transformation was done on Psychometrica [37]. For effect sizes that were transformed (OR, RR) or calculated (MD), standard error was calculated manually from the provided confidence intervals. For results originally reported in SMD, Cohen’s *d*, or Hedge’s *g*, standard error was calculated using R. A sensitivity analysis was conducted to assess the robustness of findings across effect size transformation.

All subsequent analyses were conducted using R (Details can be found in Supplementary S5). Effect sizes for each intervention type, stratified into three levels of specificity, were combined using random effects meta-analysis. Overall effects between intervention types were compared using independent-sample t-tests (where there were only two categories) and one-way ANOVAs (where there were more than two categories). Homogeneity of variances was evaluated using Levene’s test, and when this assumption was violated, Welch’s ANOVA was used. Post-hoc pairwise comparisons were performed using Tukey’s HSD test for one-way ANOVAs and the Games-Howell test for Welch’s ANOVAs when variances were unequal.

Two reviewers independently assessed the quality of each included publication using AMSTAR 2 [38]. Please refer to Supplementary S6 for full study breakdown. Results of homogeneity tests (I^2^ or Q) for primary outcomes were also extracted and coded on a binary scale (0–large heterogeneity/sign Q or I > 50%; 1–homogeneity/non-sign Q or I < 50%). Ratings were compared and collated by the first author, and disagreements were resolved by review of the publications.

## Results

Fifty-seven meta-analyses with 569 effect sizes were identified (Fig. 1). Studies were of relatively high quality but generally did not report reasons for study design or funding sources of included studies (See supplementary S6). Effect sizes (ES) were based on four [39, 40] to 101 primary studies [41] with between 127 [14] and 11,660 participants [42]. There were 357 effects for anxiety, 206 for depression, and six for combined outcomes.

The majority of reported effect sizes were for intervention-versus-control comparisons. Pre- versus post-intervention effects, measuring within-group change, were not combined with intervention-control effects, measuring between-group differences, in accordance with statistical guidance [43]. There was not enough data on pre- versus post-intervention effects in this review to report them independently [14, 43–46]. Active intervention comparisons that did not include a control condition, and which therefore provided estimates of relative efficacy, were also not combined with intervention-versus-control effects. As there was also not enough data on any given comparison to analyze, effects from three meta-analyses that reported only active intervention comparisons [47–49], and effects from seven meta-analyses which included some active treatment comparisons [19, 41, 50–54] were excluded. Therefore, final outcomes are from intervention-versus-control comparisons only.

Another 25 effects from five meta-analyses [30, 52, 55–57] could not be transformed due to lack of information and were excluded.

A threshold of four effect sizes per outcome was established a priori to ensure that pooled results were based on a sufficient number of effects for reliability and statistical power. Meta-analyses were also assessed for significant overlap in primary studies, with a threshold of > 75% overlap established a priori. Resultingly, for anxiety, six psychosocial-based meta-analyses [19, 44, 58–61] and three pharmacological-based meta-analyses were excluded [19, 54, 56]. For depression, one physical-based meta-analysis [62] was excluded and two psychosocial-based meta-analyses [30, 63] were excluded only at Level 1. These two meta-analyses (and the 10 associated effects) exceeded the threshold of 75% primary study overlap with another included meta-analysis at Level 1, but were a part of different sub-categories at Levels 2 and 3 due to increased specificity of the meta-analytic category. Therefore, they were excluded at Level 1 but included at Levels 2 and 3.

Final results of this umbrella review were based on 43 meta-analyses and 391 effect sizes (Table 1; Supplementary S7).

**Table 1:** Characteristics of included meta-analyses by effect

Authors	YoP	Country	Primary Studies	Number of Participants	Outcome	Intervention Type	Intervention Details
*Alemdar & Karaca	2025	Türkiye	29	3028	Anxiety	Psychosocial	CBT
*Alemdar & Karaca	2025	Türkiye	14	1461	Anxiety	Psychosocial	CBT
*Alemdar & Karaca	2025	Türkiye	8	835	Anxiety	Psychosocial	CBT
*Alemdar & Karaca	2025	Türkiye	4	417	Anxiety	Psychosocial	CBT
*Alemdar & Karaca	2025	Türkiye	20	2088	Anxiety	Psychosocial	CBT
*Alemdar & Karaca	2025	Türkiye	9	940	Anxiety	Psychosocial	CBT
*Alozkan-Sever et al	2023	Netherlands	19	3984	Anxiety	Psychosocial	CBT, Psychodynamic, Self-Help
*Alozkan-Sever et al	2023	Netherlands	14	2765	Anxiety	Psychosocial	CBT, Psychodynamic, Self-Help
*Alozkan-Sever et al	2023	Netherlands	26	4163	Depression	Psychosocial	CBT, IPT, Self-Help
*Alozkan-Sever et al	2023	Netherlands	23	4543	Depression	Psychosocial	CBT, IPT, Self-Help
*Alozkan-Sever et al	2023	Netherlands	14	2765	Depression	Psychosocial	CBT, IPT, Self-Help
*Alozkan-Sever et al	2023	Netherlands	12	2370	Depression	Psychosocial	CBT, IPT, Self-Help
*Alozkan-Sever et al	2023	Netherlands	12	2072	Depression	Psychosocial	CBT, IPT, Self-Help
*Alozkan-Sever et al	2023	Netherlands	10	1975	Depression	Psychosocial	CBT, IPT, Self-Help
*Alozkan-Sever et al	2023	Netherlands	4	790	Depression	Psychosocial	CBT, IPT, Self-Help
*Alozkan-Sever et al	2023	Netherlands	17	3358	Depression	Psychosocial	CBT, IPT, Self-Help
*Alozkan-Sever et al	2023	Netherlands	7	1383	Depression	Psychosocial	CBT, IPT, Self-Help
*Alozkan-Sever et al	2023	Netherlands	9	1669	Depression	Psychosocial	CBT, IPT, Self-Help
*Alozkan-Sever et al	2023	Netherlands	17	2494	Depression	Psychosocial	CBT, IPT, Self-Help
*Alozkan-Sever et al	2023	Netherlands	3	1050	Depression	Psychosocial	CBT, IPT, Self-Help
*Alozkan-Sever et al	2023	Netherlands	23	3858	Depression	Psychosocial	CBT, IPT, Self-Help
*Alozkan-Sever et al	2023	Netherlands	10	1975	Anxiety	Psychosocial	CBT, Psychodynamic, Self-Help
*Alozkan-Sever et al	2023	Netherlands	9	1778	Anxiety	Psychosocial	Psychodynamic, Self-Help
*Alozkan-Sever et al	2023	Netherlands	9	2081	Anxiety	Psychosocial	CBT, Psychodynamic, Self-Help
*Alozkan-Sever et al	2023	Netherlands	7	1383	Anxiety	Psychosocial	CBT, Psychodynamic, Self-Help
*Alozkan-Sever et al	2023	Netherlands	11	2173	Anxiety	Psychosocial	CBT, Psychodynamic, Self-Help
*Alozkan-Sever et al	2023	Netherlands	6	1185	Anxiety	Psychosocial	CBT, Psychodynamic, Self-Help
*Alozkan-Sever et al	2023	Netherlands	8	2604	Anxiety	Psychosocial	CBT, Psychodynamic, Self-Help
*Alozkan-Sever et al	2023	Netherlands	11	1380	Anxiety	Psychosocial	CBT, Psychodynamic, Self-Help
*Alozkan-Sever et al	2023	Netherlands	5	1138	Anxiety	Psychosocial	CBT, Psychodynamic, Self-Help
*Alozkan-Sever et al	2023	Netherlands	14	3486	Anxiety	Psychosocial	CBT, Psychodynamic, Self-Help
*Alozkan-Sever et al	2023	Netherlands	23	3984	Anxiety	Psychosocial	CBT, Psychodynamic, Self-Help
*Alozkan-Sever et al	2023	Netherlands	26	4163	Depression	Psychosocial	CBT, Psychodynamic, Self-Help
*Alozkan-Sever et al	2023	Netherlands	7	11,660	Anxiety	Psychosocial	CBT, Psychodynamic, Self-Help
Arnardóttir & Skarphedinsson	2022	Iceland	78	5844	Anxiety	Psychosocial	CBT
Arnardóttir & Skarphedinsson	2022	Iceland	30	3442	Anxiety	Pharmacological	SG Antidepressants
Arnardóttir & Skarphedinsson	2022	Iceland	86	3797	Anxiety	Psychosocial	CBT
Arnardóttir & Skarphedinsson	2022	Iceland	78	5049	Anxiety	Psychosocial	CBT
Arnardóttir & Skarphedinsson	2022	Iceland	8	1395	Anxiety	Pharmacological	SG Antidepressants
Arnardóttir & Skarphedinsson	2022	Iceland	56	2647	Anxiety	Pharmacological	SG Antidepressants
Arnardóttir & Skarphedinsson	2022	Iceland	78	5844	Anxiety	Psychosocial	CBT
Arnardóttir & Skarphedinsson	2022	Iceland	30	3442	Anxiety	Pharmacological	SG Antidepressants
Arnardóttir & Skarphedinsson	2022	Iceland	86	3797	Anxiety	Psychosocial	CBT
Arnardóttir & Skarphedinsson	2022	Iceland	78	5049	Anxiety	Psychosocial	CBT
Arnardóttir & Skarphedinsson	2022	Iceland	8	1395	Anxiety	Pharmacological	SG Antidepressants
Arnardóttir & Skarphedinsson	2022	Iceland	56	2647	Anxiety	Pharmacological	SG Antidepressants
Axelsdottir et al	2021	Norway	4	159	Depression	Physical	Exercise
Baker et al	2021	UK	16	766	Anxiety	Psychosocial	CBT, 1 other
Baker et al	2021	UK	9	563	Anxiety	Psychosocial	CBT
Baker et al	2021	UK	9	421	Anxiety	Psychosocial	CBT
Baker et al	2021	UK	9	388	Anxiety	Psychosocial	CBT
Baker et al	2021	UK	4	257	Anxiety	Psychosocial	CBT
Baker et al	2021	UK	4	285	Anxiety	Psychosocial	CBT (computer)
Baker et al	2021	UK	16	766	Anxiety	Psychosocial	CBT
Baker et al	2021	UK	16	766	Anxiety	Psychosocial	CBT
Baker et al	2021	UK	9	279	Anxiety	Psychosocial	CBT
Baker et al	2021	UK	5	360	Anxiety	Psychosocial	CBT
Baourda et al	2022	Greece	12	1132	Anxiety	Psychosocial	CBT, psycho-education, skills, play
Baourda et al	2022	Greece	11	933	Anxiety	Psychosocial	CBT, psycho-education, skills, play
Campisi et al	2021	Canada	7	490	Depression	Physical	Food and exercise
*Carneiro et al	2025	Portugal	7	11,660	Depression	Psychosocial	MBI
*Carneiro et al	2025	Portugal	8	9522	Anxiety	Psychosocial	MBI
*Carneiro et al	2025	Portugal	5	11,337	Depression	Psychosocial	MBI
*Carneiro et al	2025	Portugal	5	9073	Anxiety	Psychosocial	MBI
Cervin & Lundgren	2021	Sweden	9	711	Anxiety	Psychosocial	CBT
Cervin & Lundgren	2021	Sweden	8	690	Anxiety	Psychosocial	CBT
Cervin & Lundgren	2021	Sweden	9	655	Anxiety	Psychosocial	CBT
Cervin & Lundgren	2021	Sweden	7	590	Anxiety	Psychosocial	CBT
Cervin & Lundgren	2021	Sweden	7	572	Anxiety	Psychosocial	CBT
*Csirmaz et al	2024	Hungary	18	1262	Anxiety	Psychosocial	CBT, bibliotherapy, Family Intervention
*Csirmaz et al	2024	Hungary	12	870	Anxiety	Psychosocial	CBT, bibliotherapy, Family Intervention
*Csirmaz et al	2024	Hungary	15	1101	Anxiety	Psychosocial	CBT, bibliotherapy, Family Intervention
Cuijpers et al	2021	Netherlands	12 (13)	1576	Depression	Psychosocial	CBT, IPT, SUP
Cuijpers et al	2021	Netherlands	9	1091	Depression	Psychosocial	CBT
Cuijpers et al	2021	Netherlands	4	485	Depression	Psychosocial	IPT, SUP
Cuijpers et al	2021	Netherlands	11	1334	Depression	Psychosocial	CBT, IPT, SUP
Cuijpers et al	2021	Netherlands	2	242	Depression	Psychosocial	CBT, IPT, SUP
*Cuijpers et al	2023	Netherlands	38	3779	Depression	Psychosocial	CBT, IPT, Other
*Cuijpers et al	2023	Netherlands	32	3680	Depression	Psychosocial	CBT, IPT, Other
*Cuijpers et al	2023	Netherlands	12	1193	Depression	Psychosocial	CBT, IPT, Other
*Cuijpers et al	2023	Netherlands	12	895	Depression	Psychosocial	CBT, IPT, Other
*Cuijpers et al	2023	Netherlands	38	3779	Depression	Psychosocial	CBT, IPT, Other
*Cuijpers et al	2023	Netherlands	38	3779	Depression	Psychosocial	CBT, IPT, Other
*Cuijpers et al	2023	Netherlands	6	796	Depression	Psychosocial	CBT, IPT, Other
Dippel et al	2022	Germany	17	1208	Depression	Psychosocial	Family Intervention
Dobson et al	2019	USA	8	853	Anxiety	Pharmacological	SG Antidepressants
Dobson et al	2019	USA	5	990	Anxiety	Pharmacological	SG Antidepressants
Dobson et al	2019	USA	8	853	Anxiety	Pharmacological	SG Antidepressants
Dobson et al	2019	USA	5	990	Anxiety	Pharmacological	SG Antidepressants
Dobson et al	2019	USA	5	144	Anxiety	Pharmacological	FG Antidepressants
Eckshtain et al	2022	USA	11	1004	Depression	Psychosocial	Family Intervention
Eckshtain et al	2022	USA	11	1004	Depression	Psychosocial	Family Intervention
Eckshtain et al	2022	USA	8	730	Depression	Psychosocial	Family Intervention
Eckshtain et al	2022	USA	7	639	Depression	Psychosocial	Family Intervention
Eckshtain et al	2022	USA	4	365	Depression	Psychosocial	Family Intervention
Eckshtain et al	2020	USA	20	1691	Depression	Psychosocial	CBT, IPT
Eckshtain et al	2020	USA	53	4394	Depression	Psychosocial	CBT, IPT
Eckshtain et al	2020	USA	30	2705	Depression	Psychosocial	CBT, IPT
Eckshtain et al	2020	USA	30	2536	Depression	Psychosocial	CBT, IPT
Eckshtain et al	2020	USA	167	2705	Depression	Psychosocial	CBT, IPT
Eckshtain et al	2020	USA	188	3297	Depression	Psychosocial	CBT, IPT
Eckshtain et al	2020	USA	34	1184	Depression	Psychosocial	CBT, IPT
Eckshtain et al	2020	USA	177	3382	Depression	Psychosocial	CBT, IPT
Eckshtain et al	2020	USA	30	845	Depression	Psychosocial	CBT, IPT
Eckshtain et al	2020	USA	129	2283	Depression	Psychosocial	CBT, IPT
Eckshtain et al	2021	USA	43	1099	Depression	Psychosocial	CBT, IPT
Eckshtain et al	2022	USA	49	1353	Depression	Psychosocial	CBT, IPT
Eckshtain et al	2023	USA	173	3128	Depression	Psychosocial	CBT, IPT
Eckshtain et al	2024	USA	64	1522	Depression	Psychosocial	CBT, IPT
Eckshtain et al	2025	USA	152	2875	Depression	Psychosocial	CBT, IPT
Eckshtain et al	2026	USA	97	1522	Depression	Psychosocial	CBT, IPT
Eckshtain et al	2027	USA	50	1353	Depression	Psychosocial	CBT, IPT
Eckshtain et al	2028	USA	31	676	Depression	Psychosocial	CBT, IPT
Eckshtain et al	2029	USA	103	2536	Depression	Psychosocial	CBT, IPT
Eckshtain et al	2030	USA	158	4312	Depression	Psychosocial	CBT, IPT
Eckshtain et al	2031	USA	25	1015	Depression	Psychosocial	CBT, IPT
Eckshtain et al	2032	USA	38	1353	Depression	Psychosocial	CBT, IPT
Eckshtain et al	2033	USA	68	1353	Depression	Psychosocial	CBT, IPT
Eckshtain et al	2034	USA	107	2452	Depression	Psychosocial	CBT, IPT
Eckshtain et al	2035	USA	28	592	Depression	Psychosocial	CBT, IPT
Eckshtain et al	2036	USA	137	2875	Depression	Psychosocial	CBT
Eckshtain et al	2037	USA	24	423	Depression	Psychosocial	IPT
Eckshtain et al	2038	USA	26	592	Depression	Psychosocial	CBT
Eckshtain et al	2039	USA	114	2367	Depression	Psychosocial	CBT, IPT
Eckshtain et al	2040	USA	47	1015	Depression	Psychosocial	CBT, IPT
Eckshtain et al	2041	USA	54	1268	Depression	Psychosocial	CBT, IPT
Eckshtain et al	2020	USA	28	2321	Depression	Psychosocial	CBT, IPT
Eckshtain et al	2020	USA	25	2073	Depression	Psychosocial	CBT, IPT
Fulambarkar et al	2022	USA	4	837	Anxiety	Psychosocial	MBI
Fulambarkar et al	2022	USA	6	3172	Depression	Psychosocial	MBI
Hang et al	2021	China	14	749	Anxiety	Psychosocial	Bias modification (ABM)
Hang et al	2021	China	13	686	Anxiety	Psychosocial	Bias modification (ABM)
Hang et al	2021	China	11	586	Anxiety	Psychosocial	Bias modification (ABM)
Hang et al	2021	China	8	466	Anxiety	Psychosocial	Bias modification (ABM)
Hang et al	2021	China	4	189	Anxiety	Psychosocial	Bias modification (ABM)
Hang et al	2021	China	4	171	Anxiety	Psychosocial	Bias modification (ABM)
Hang et al	2021	China	9	515	Anxiety	Psychosocial	Bias modification (ABM)
Hang et al	2021	China	7	330	Anxiety	Psychosocial	Bias modification (ABM)
Hang et al	2021	China	6	356	Anxiety	Psychosocial	Bias modification (ABM)
Hetrick et al	2021	UK	5	1307	Depression	Pharmacological	SG Antidepressants, Fluoxetine
Hetrick et al	2021	UK	4	1045	Depression	Pharmacological	SG Antidepressants, Paroxetine
Hetrick et al	2021	UK	5	1307	Depression	Pharmacological	SG Antidepressants, Fluoxetine
Hetrick et al	2021	UK	4	1045	Depression	Pharmacological	SG Antidepressants, Paroxetine
Hetrick et al	2021	UK	21	5227	Depression	Pharmacological	SG Antidepressants
Hetrick et al	2021	UK	5	1307	Depression	Pharmacological	SG Antidepressants
Hetrick et al	2021	UK	21	5227	Depression	Pharmacological	SG Antidepressants
Hetrick et al	2021	UK	5	1307	Depression	Pharmacological	SG Antidepressants
Higinbotham	2020	USA	7	662	Depression	Psychosocial	CBT (BITS)
Higinbotham	2020	USA	4	459	Depression	Psychosocial	CBT (BITS)
Higinbotham	2020	USA	12	2174	Depression	Psychosocial	CBT (a)
Higinbotham	2020	USA	4	819	Depression	Psychosocial	CBT (F2F)
Higinbotham	2020	USA	5	379	Depression	Psychosocial	CBT (F2F)
Higinbotham	2020	USA	7	522	Depression	Psychosocial	CBT (F2F)
Higinbotham	2020	USA	4	478	Depression	Psychosocial	CBT (BITS)
Higinbotham	2020	USA	12	1786	Depression	Psychosocial	CBT (BITS)
Higinbotham	2020	USA	7	1030	Depression	Psychosocial	CBT (BITS)
Hugh-Jones et al	2020	UK	21	1765	Anxiety	Psychosocial	CBT, play, MBI, others
Hugh-Jones et al	2020	UK	9	1297	Anxiety	Psychosocial	CBT, play, MBI, others
Hugh-Jones et al	2020	UK	8	824	Anxiety	Psychosocial	CBT, play, MBI, others
Hugh-Jones et al	2020	UK	7	644	Anxiety	Psychosocial	CBT
Hugh-Jones et al	2020	UK	4	457	Anxiety	Psychosocial	CBT
Hugh-Jones et al	2020	UK	7	460	Anxiety	Psychosocial	CBT
Hugh-Jones et al	2020	UK	5	490	Anxiety	Psychosocial	CBT, play, MBI, others
Hugh-Jones et al	2020	UK	4	392	Anxiety	Psychosocial	CBT, play, MBI, others
Hugh-Jones et al	2020	UK	11	1079	Anxiety	Psychosocial	CBT, play, MBI, others
James et al	2020	UK	39	2697	Anxiety	Psychosocial	CBT
James et al	2020	UK	23	1184	Anxiety	Psychosocial	CBT
James et al	2020	UK	20	1142	Anxiety	Psychosocial	CBT
James et al	2020	UK	5	371	Anxiety	Psychosocial	CBT
James et al	2020	UK	19	1165	Anxiety	Psychosocial	CBT
James et al	2020	UK	30	1532	Anxiety	Psychosocial	CBT
James et al	2020	UK	8	513	Anxiety	Psychosocial	CBT
James et al	2020	UK	27	1622	Anxiety	Psychosocial	CBT
James et al	2020	UK	13	562	Anxiety	Psychosocial	CBT
James et al	2020	UK	13	789	Anxiety	Psychosocial	CBT
James et al	2020	UK	26	1447	Anxiety	Psychosocial	CBT
James et al	2020	UK	9	461	Anxiety	Psychosocial	CBT
James et al	2020	UK	44	2561	Anxiety	Psychosocial	CBT
James et al	2020	UK	47	2406	Anxiety	Psychosocial	CBT
James et al	2020	UK	57	3158	Anxiety	Psychosocial	CBT
James et al	2020	UK	28	2075	Anxiety	Psychosocial	CBT
James et al	2020	UK	12	564	Anxiety	Psychosocial	CBT
James et al	2020	UK	15	916	Anxiety	Psychosocial	CBT
James et al	2020	UK	8	595	Anxiety	Psychosocial	CBT
James et al	2020	UK	11	671	Anxiety	Psychosocial	CBT
James et al	2020	UK	24	1404	Anxiety	Psychosocial	CBT
James et al	2020	UK	6	461	Anxiety	Psychosocial	CBT
James et al	2020	UK	19	1123	Anxiety	Psychosocial	CBT
James et al	2020	UK	10	491	Anxiety	Psychosocial	CBT
James et al	2020	UK	12	950	Anxiety	Psychosocial	CBT
James et al	2020	UK	20	980	Anxiety	Psychosocial	CBT
James et al	2020	UK	34	1871	Anxiety	Psychosocial	CBT
James et al	2020	UK	45	2831	Anxiety	Psychosocial	CBT
James et al	2020	UK	29	1239	Anxiety	Psychosocial	CBT
James et al	2020	UK	22	1285	Anxiety	Psychosocial	CBT
James et al	2020	UK	6	307	Anxiety	Psychosocial	CBT
James et al	2020	UK	49	2459	Anxiety	Psychosocial	CBT
James et al	2020	UK	25	1203	Anxiety	Psychosocial	CBT
James et al	2020	UK	32	1628	Anxiety	Psychosocial	CBT
James et al	2020	UK	12	587	Anxiety	Psychosocial	CBT
James et al	2020	UK	33	1840	Anxiety	Psychosocial	CBT
James et al	2020	UK	10	404	Anxiety	Psychosocial	CBT
James et al	2020	UK	12	663	Anxiety	Psychosocial	CBT
James et al	2020	UK	38	1834	Anxiety	Psychosocial	CBT
James et al	2020	UK	7	334	Anxiety	Psychosocial	CBT
James et al	2020	UK	52	2650	Anxiety	Psychosocial	CBT
James et al	2020	UK	35	2137	Anxiety	Psychosocial	CBT
James et al	2020	UK	17	734	Anxiety	Psychosocial	CBT
James et al	2020	UK	18	1031	Anxiety	Psychosocial	CBT
James et al	2020	UK	6	372	Anxiety	Psychosocial	CBT
James et al	2020	UK	37	1952	Anxiety	Psychosocial	CBT
James et al	2020	UK	18	858	Anxiety	Psychosocial	CBT
James et al	2020	UK	23	1279	Anxiety	Psychosocial	CBT
James et al	2020	UK	5	336	Anxiety	Psychosocial	CBT
James et al	2020	UK	26	1402	Anxiety	Psychosocial	CBT
James et al	2020	UK	10	399	Anxiety	Psychosocial	CBT
James et al	2020	UK	12	750	Anxiety	Psychosocial	CBT
James et al	2020	UK	22	1057	Anxiety	Psychosocial	CBT
James et al	2020	UK	7	330	Anxiety	Psychosocial	CBT
James et al	2020	UK	34	1893	Anxiety	Psychosocial	CBT
James et al	2020	UK	23	1157	Anxiety	Psychosocial	CBT
James et al	2020	UK	22	1476	Anxiety	Psychosocial	CBT
James et al	2020	UK	37	2037	Anxiety	Psychosocial	CBT
James et al	2020	UK	8	487	Anxiety	Psychosocial	CBT
James et al	2021	UK	5	172	Anxiety	Psychosocial	CBT
James et al	2023	UK	4	345	Anxiety	Psychosocial	CBT
James et al	2023	UK	8	440	Anxiety	Psychosocial	CBT
James et al	2020	UK	4	151	Anxiety	Psychosocial	CBT
James et al	2020	UK	8	441	Anxiety	Psychosocial	CBT
James et al	2020	UK	5	203	Anxiety	Psychosocial	CBT
James et al	2020	UK	6	214	Anxiety	Psychosocial	CBT
James et al	2020	UK	5	187	Anxiety	Psychosocial	CBT
James et al	2020	UK	7	228	Anxiety	Psychosocial	CBT
James et al	2020	UK	6	202	Anxiety	Psychosocial	CBT
James et al	2020	UK	10	822	Anxiety	Psychosocial	CBT
James et al	2020	UK	9	509	Anxiety	Psychosocial	CBT
James et al	2021	UK	4	313	Anxiety	Psychosocial	CBT
James et al	2022	UK	6	469	Anxiety	Psychosocial	CBT
James et al	2023	UK	7	353	Anxiety	Psychosocial	CBT
James et al	2024	UK	13	752	Anxiety	Psychosocial	CBT
James et al	2024	UK	6	341	Anxiety	Psychosocial	CBT
James et al	2020	UK	7	378	Anxiety	Psychosocial	CBT
James et al	2020	UK	7	359	Anxiety	Psychosocial	CBT
James et al	2020	UK	21	978	Anxiety	Psychosocial	CBT
James et al	2020	UK	14	685	Anxiety	Psychosocial	CBT
James et al	2020	UK	9	434	Anxiety	Psychosocial	CBT
James et al	2020	UK	12	638	Anxiety	Psychosocial	CBT
James et al	2020	UK	5	345	Anxiety	Psychosocial	CBT
James et al	2020	UK	9	434	Anxiety	Psychosocial	CBT
James et al	2020	UK	4	401	Anxiety	Psychosocial	CBT
James et al	2020	UK	4	348	Anxiety	Psychosocial	CBT
James et al	2020	UK	6	399	Anxiety	Psychosocial	CBT
James et al	2020	UK	5	378	Anxiety	Psychosocial	CBT
James et al	2020	UK	6	423	Anxiety	Psychosocial	CBT
James et al	2020	UK	4	153	Anxiety	Psychosocial	CBT
James et al	2020	UK	6	179	Anxiety	Psychosocial	CBT
James et al	2020	UK	5	201	Anxiety	Psychosocial	CBT
James et al	2020	UK	6	302	Anxiety	Psychosocial	CBT
James et al	2020	UK	13	613	Depression	Psychosocial	CBT
Jewell et al	2022	UK	9	690	Anxiety	Psychosocial	Family Intervention (parent-only, CBT)
Jewell et al	2022	UK	7	621	Anxiety	Psychosocial	Family Intervention (parent-only, CBT)
Keles & Idsoe	2018	Norway	49	5469	Depression	Psychosocial	CBT
Keles & Idsoe	2018	Norway	56	4042	Depression	Psychosocial	CBT
Krebs et al	2017	UK	17	1168	Anxiety	Psychosocial	Bias modification (CBM)
Krebs et al	2017	UK	7	481	Anxiety	Psychosocial	Bias modification (CBM)
Kreuze et al	2018	Netherlands	31	2326	Anxiety	Psychosocial	CBT
Kreuze et al	2018	Netherlands	42	3239	Anxiety	Psychosocial	CBT
Kreuze et al	2018	Netherlands	33	2545	Anxiety	Psychosocial	CBT
Kreuze et al	2018	Netherlands	11	900	Anxiety	Psychosocial	CBT
Kreuze et al	2018	Netherlands	16	1365	Anxiety	Psychosocial	CBT
Kreuze et al	2018	Netherlands	22	1434	Anxiety	Psychosocial	CBT
Kreuze et al	2018	Netherlands	24	2023	Anxiety	Psychosocial	CBT
*Li et al	2023	China	35	5393	Depression	Physical	Exercise
*Li et al	2023	China	20	3082	Depression	Physical	Exercise
*Li et al	2023	China	14	2157	Depression	Physical	Exercise
*Li et al	2023	China	2	308	Depression	Physical	Exercise
*Li et al	2023	China	5	770	Depression	Physical	Exercise
*Li et al	2023	China	17	2619	Depression	Physical	Exercise
*Li et al	2023	China	11	1695	Depression	Physical	Exercise
*Li et al	2023	China	7	1079	Depression	Physical	Exercise
*Li et al	2023	China	13	2003	Depression	Physical	Exercise
*Li et al	2023	China	7	1079	Depression	Physical	Exercise
*Li et al	2023	China	5	770	Depression	Physical	Exercise
*Li et al	2023	China	5	770	Depression	Physical	Exercise
*Li et al	2023	China	6	925	Depression	Physical	Exercise
*Li et al	2023	China	14	2157	Depression	Physical	Exercise
*Li et al	2023	China	6	925	Depression	Physical	Exercise
*Li et al	2023	China	16	2465	Depression	Physical	Exercise
*Li et al	2023	China	8	1233	Depression	Physical	Exercise
*Li et al	2025	China	6	172	Anxiety	Psychosocial	ACT
*Li et al	2025	China	16	969	Anxiety	Psychosocial	CBT
*Li et al	2025	China	6	242	Anxiety	Psychosocial	Virtual Reality Exposure Therapy
*Liu et al	2024	China	16	1219	Depression	Psychosocial	CBT
*Liu et al	2024	China	10	1810	Depression	Psychosocial	CBT
*Liu et al	2024	China	4	544	Depression	Psychosocial	CBT
*Liu et al	2024	China	7	562	Depression	Psychosocial	CBT
*Liu et al	2024	China	4	286	Depression	Psychosocial	CBT
* Lopez-Pinar et al	2025	Spain	12	445	Depression	Psychosocial	ACT
* Lopez-Pinar et al	2025	Spain	5	142	Depression	Psychosocial	ACT
* Lopez-Pinar et al	2025	Spain	15	1368	Depression	Psychosocial	ACT
* Lopez-Pinar et al	2025	Spain	6	558	Depression	Psychosocial	ACT
* Lopez-Pinar et al	2025	Spain	10	530	Anxiety	Psychosocial	ACT
* Lopez-Pinar et al	2025	Spain	5	222	Anxiety	Psychosocial	ACT
* Lopez-Pinar et al	2025	Spain	15	1325	Anxiety	Psychosocial	ACT
* Lopez-Pinar et al	2025	Spain	7	658	Anxiety	Psychosocial	ACT
Luo & McAloon	2021	Australia	21	1014	Anxiety	Psychosocial	CBT
Luo & McAloon	2021	Australia	17	596	Anxiety	Psychosocial	CBT
Luo & McAloon	2021	Australia	10	477	Anxiety	Psychosocial	CBT
Luo & McAloon	2021	Australia	15	537	Anxiety	Psychosocial	CBT
Oberste et al	2020	Germany	12	431	Depression	Physical	Exercise
Oberste et al	2020	Germany	7	307	Depression	Physical	Exercise
Oberste et al	2020	Germany	8	329	Depression	Physical	Exercise
Oberste et al	2020	Germany	4	174	Depression	Physical	Exercise
Oberste et al	2020	Germany	5	127	Depression	Physical	Exercise
Oberste et al	2020	Germany	9	301	Depression	Physical	Exercise
Oberste et al	2020	Germany	9	267	Depression	Physical	Exercise
Oberste et al	2020	Germany	6	196	Depression	Physical	Exercise
Oberste et al	2020	Germany	6	235	Depression	Physical	Exercise
Odgers et al	2020	Australia	20	1582	Anxiety	Psychosocial	MBI
Odgers et al	2020	Australia	7	978	Anxiety	Psychosocial	MBI
Odgers et al	2020	Australia	7	270	Anxiety	Psychosocial	MBI
Odgers et al	2020	Australia	13	1312	Anxiety	Psychosocial	MBI
Odgers et al	2020	Australia	6	305	Anxiety	Psychosocial	MBI
Odgers et al	2020	Australia	12	1244	Anxiety	Psychosocial	MBI
Odgers et al	2020	Australia	8	395	Anxiety	Psychosocial	MBI
Odgers et al	2020	Australia	5	324	Anxiety	Psychosocial	MBI
Odgers et al	2020	Australia	15	1280	Anxiety	Psychosocial	MBI
Odgers et al	2020	Australia	5	1280	Anxiety	Psychosocial	MBI
Pu et al	2017	China	7	538	Depression	Psychosocial	IPT
Pu et al	2017	China	4	367	Depression	Psychosocial	IPT
Pu et al	2017	China	4	235	Depression	Psychosocial	IPT
Pu et al	2017	China	4	231	Depression	Psychosocial	IPT
Pu et al	2017	China	4	231	Depression	Psychosocial	IPT
Pu et al	2017	China	6	465	Depression	Psychosocial	IPT
Reyad et al	2021	UK	6	1193	Depression	Pharmacological	SG Antidepressants, Fluoxetine
Reyad et al	2021	UK	5	972	Depression	Pharmacological	SG Antidepressants, Fluoxetine
Schwartz et al	2019	Canada	14	1421	Anxiety	Psychosocial	CBT
Schwartz et al	2019	Canada	11	1235	Anxiety	Pharmacological	SG Antidepressants
Schwartz et al	2019	Canada	14	1014	Anxiety	Psychosocial	CBT
Steains et al	2021	Australia	5	233	Anxiety	Psychosocial	Behavioral systems
Steains et al	2021	Australia	5	233	Anxiety	Psychosocial	Behavioral systems
Steains et al	2021	Australia	5	233	Anxiety	Psychosocial	Behavioral systems
Stefánsdóttir et al	2022	Iceland	6	913	Anxiety	Pharmacological	SG Antidepressants
Stefánsdóttir et al	2022	Iceland	11	1774	Anxiety	Pharmacological	SG Antidepressants
Stefánsdóttir et al	2022	Iceland	11	1783	Anxiety	Pharmacological	SG Antidepressants
Stefánsdóttir et al	2022	Iceland	9	1961	Anxiety	Pharmacological	SG Antidepressants
Strawn et al	2018	USA	9	1805	Anxiety	Pharmacological	SG Antidepressants
Tejada-Gallardo	2020	Spain	4	1694	Anxiety	Psychosocial	Positive Psychology
Tejada-Gallardo	2020	Spain	4	1694	Depression	Psychosocial	Positive Psychology
Teng et al	2022	China	5	272	Depression	Pharmacological	FG Antidepressants
Teng et al	2022	China	13	2327	Depression	Pharmacological	SG Antidepressants
Teng et al	2022	China	5	526	Depression	Pharmacological	SG Antidepressants, Fluoxetine
Teng et al	2022	China	8	1167	Depression	Pharmacological	Antidepressants
Teng et al	2022	China	7	295	Depression	Pharmacological	Antidepressants
Teng et al	2022	China	10	2242	Depression	Pharmacological	Antidepressants
Teng et al	2022	China	8	638	Depression	Pharmacological	Antidepressants
Teng et al	2022	China	9	1899	Depression	Pharmacological	Antidepressants
Teng et al	2022	China	13	1596	Depression	Pharmacological	Antidepressants
Teng et al	2022	China	4	941	Depression	Pharmacological	Antidepressants
Teng et al	2022	China	10	2078	Depression	Pharmacological	Antidepressants
Teng et al	2022	China	7	459	Depression	Pharmacological	Antidepressants
*Tindall et al	2024	UK	4	156	Depression	Psychosocial	BA
*van Aswegen et al	2023	Netherlands	4	549	Depression	Psychosocial	Family Intervention
*van Aswegen et al	2023	Netherlands	8	1097	Depression	Psychosocial	Family Intervention
*van Aswegen et al	2023	Netherlands	4	549	Depression	Psychosocial	Family Intervention
*van Aswegen et al	2023	Netherlands	5	686	Depression	Psychosocial	Family Intervention
*Waraan et al	2023	Norway	9	788	Depression	Psychosocial	Family Intervention
Wang et al	2022	China	15 (19)	1331	Depression	Physical	Exercise
Wang et al	2022	China	6	251	Depression	Physical	Exercise
Wang et al	2022	China	13	1080	Depression	Physical	Exercise
Whiteside et al	2020	USA	19	926	Anxiety	Psychosocial	CBT
Whiteside et al	2020	USA	27	1316	Anxiety	Psychosocial	CBT
Whiteside et al	2020	USA	15	731	Anxiety	Psychosocial	CBT
Whiteside et al	2020	USA	19	926	Anxiety	Psychosocial	CBT
Whiteside et al	2020	USA	27	1316	Anxiety	Psychosocial	CBT
Whiteside et al	2020	USA	14	683	Anxiety	Psychosocial	CBT
Whiteside et al	2020	USA	19	926	Anxiety	Psychosocial	CBT
Whiteside et al	2020	USA	27	1316	Anxiety	Psychosocial	CBT
Whiteside et al	2020	USA	15	731	Anxiety	Psychosocial	CBT
Whiteside et al	2020	USA	19	926	Anxiety	Psychosocial	CBT
Whiteside et al	2020	USA	27	1316	Anxiety	Psychosocial	CBT
Whiteside et al	2020	USA	15	731	Anxiety	Psychosocial	CBT
Whiteside et al	2020	USA	19	926	Anxiety	Psychosocial	CBT
Whiteside et al	2020	USA	27	1316	Anxiety	Psychosocial	CBT
Whiteside et al	2020	USA	15	731	Anxiety	Psychosocial	CBT
Whiteside et al	2020	USA	18	878	Anxiety	Psychosocial	CBT
Whiteside et al	2020	USA	27	1316	Anxiety	Psychosocial	CBT
Whiteside et al	2020	USA	13	634	Anxiety	Psychosocial	CBT
Whiteside et al	2020	USA	19	926	Anxiety	Psychosocial	CBT
Whiteside et al	2020	USA	27	1316	Anxiety	Psychosocial	CBT
Whiteside et al	2020	USA	15	731	Anxiety	Psychosocial	CBT
Whiteside et al	2020	USA	19	926	Anxiety	Psychosocial	CBT, Family Intervention
Whiteside et al	2020	USA	27	1316	Anxiety	Psychosocial	CBT, Family Intervention
Whiteside et al	2020	USA	15	731	Anxiety	Psychosocial	CBT, Family Intervention
Yang et al	2019	China	17	1016	Anxiety	Psychosocial	CBT, BT, skills, exposure, etc
Yang et al	2019	China	8	299	Anxiety	Psychosocial	CBT, BT, skills, exposure, etc
Yang et al	2019	China	17	983	Anxiety	Psychosocial	CBT, BT, skills, exposure, etc
Yang et al	2019	China	13	832	Anxiety	Psychosocial	CBT, BT, skills, exposure, etc
Yang et al	2019	China	14	882	Anxiety	Psychosocial	CBT, BT, skills, exposure, etc
Yang et al	2019	China	13	713	Anxiety	Psychosocial	CBT, BT, skills, exposure, etc
Yang et al	2019	China	14	872	Anxiety	Psychosocial	CBT
Yang et al	2019	China	4	169	Anxiety	Psychosocial	BT
Yang et al	2019	China	11	603	Anxiety	Psychosocial	CBT, BT, skills, exposure, etc
Yang et al	2019	China	7	433	Anxiety	Psychosocial	CBT, BT, skills, exposure, etc
Yang et al	2019	China	4	303	Anxiety	Psychosocial	CBT (group)
Yang et al	2019	China	7	395	Anxiety	Psychosocial	CBT, BT, skills, exposure, etc
Yang et al	2019	China	11	670	Anxiety	Psychosocial	CBT, BT, skills, exposure, etc
Yang et al	2019	China	6	406	Anxiety	Psychosocial	CBT, BT, skills, exposure, etc
Yang et al	2019	China	11	610	Anxiety	Psychosocial	CBT, BT, skills, exposure, etc
Yang et al	2019	China	9	760	Anxiety	Psychosocial	CBT, BT, skills, exposure, etc
Yang et al	2019	China	8	256	Anxiety	Psychosocial	CBT, BT, skills, exposure, etc
Yang et al	2017	China	11	306	Depression	Psychosocial	CBT
Yang et al	2017	China	7	226	Depression	Psychosocial	CBT
Yang et al	2017	China	5	133	Depression	Psychosocial	CBT
Yang et al	2017	China	8	241	Depression	Psychosocial	CBT
Yang et al	2017	China	9	287	Depression	Psychosocial	CBT
Yang et al	2017	China	7	221	Depression	Psychosocial	CBT
*Yang et al	2025	China	7	375	Depression	Physical	Exercise
*Zhang et al	2022	China	9	433	Depression	Physical	Exercise
*Zhang et al	2022	China	6	231	Depression	Physical	Exercise
*Zhang et al	2022	China	7	202	Depression	Physical	Exercise
*Zhang et al	2022	China	6	190	Depression	Physical	Exercise
*Zhang et al	2022	China	7	243	Depression	Physical	Exercise
*Zhang et al	2022	China	5	167	Depression	Physical	Exercise
*Zhang et al	2022	China	5	130	Depression	Physical	Exercise
*Zhang et al	2022	China	9	225	Depression	Physical	Exercise
*Zhang et al	2022	China	4	208	Depression	Physical	Exercise
*Zhang et al	2022	China	7	209	Depression	Physical	Exercise
*Zhang et al	2022	China	6	226	Depression	Physical	Exercise
*Zhang et al	2022	China	5	241	Depression	Physical	Exercise
*Zhang et al	2022	China	8	192	Depression	Physical	Exercise
*Zhang et al	2022	China	9	290	Depression	Physical	Exercise
*Zhang et al	2022	China	4	143	Depression	Physical	Exercise
*Zhang et al	2025	China	22	4848	Depression	Pharmacological	Antidepressants Mixed Antidepressants Mixed Antidepressants Mixed Antidepressants
*Zhang et al	2025	China	8	2162	Depression	Pharmacological	Antidepressants
*Zhang et al	2025	China	19	4735	Depression	Pharmacological	Antidepressants
*Zhang et al	2025	China	20	4634	Depression	Pharmacological	Antidepressants
Zhou et al	2018	China	27	1040	Anxiety	Psychosocial	CBT
Zhou et al	2018	China	27	784	Anxiety	Psychosocial	CBT
Zhou et al	2018	China	27	707	Anxiety	Psychosocial	CBT (group)
Zhou et al	2018	China	53	2011	Anxiety	Psychosocial	CBT (group)
Zhou et al	2018	China	6	324	Anxiety	Psychosocial	Family Intervention (parent-only, CBT)
Zhou et al	2018	China	12	546	Anxiety	Psychosocial	Family Intervention (parent-only, CBT)
Zhou et al	2018	China	47	1443	Anxiety	Psychosocial	Family Intervention (parent-only, CBT)
Zhou et al	2018	China	30	1263	Anxiety	Psychosocial	CBT
Zhou et al	2018	China	30	1007	Anxiety	Psychosocial	CBT
Zhou et al	2018	China	30	930	Anxiety	Psychosocial	CBT
Zhou et al	2018	China	53	2234	Anxiety	Psychosocial	CBT
Zhou et al	2018	China	11	523	Anxiety	Psychosocial	CBT (Internet)
Zhou et al	2018	China	12	745	Anxiety	Psychosocial	CBT (Internet)
Zhou et al	2018	China	47	1642	Anxiety	Psychosocial	CBT (Internet)
Zhou et al	2018	China	21	1285	Anxiety	Psychosocial	CBT (group), Family Intervention (CBT)
Zhou et al	2018	China	21	1029	Anxiety	Psychosocial	CBT (group), Family Intervention (CBT)
Zhou et al	2018	China	21	952	Anxiety	Psychosocial	CBT (group), Family Intervention (CBT)
Zhou et al	2018	China	53	2256	Anxiety	Psychosocial	CBT (group), Family Intervention (CBT)
Zhou et al	2018	China	20	673	Anxiety	Psychosocial	CBT
Zhou et al	2018	China	6	480	Anxiety	Psychosocial	CBT (bibliotherapy)
Zhou et al	2018	China	17	866	Anxiety	Psychosocial	CBT (group), Family Intervention (CBT)
Zhou et al	2018	China	26	892	Anxiety	Psychosocial	CBT
Zhou et al	2018	China	24	1008	Anxiety	Psychosocial	CBT, Family Intervention
Zhou et al	2018	China	29	1422	Anxiety	Psychosocial	CBT, Family Intervention
Zhou et al	2018	China	29	1166	Anxiety	Psychosocial	CBT, Family Intervention
Zhou et al	2018	China	29	1089	Anxiety	Psychosocial	CBT, Family Intervention
Zhou et al	2018	China	53	2393	Anxiety	Psychosocial	CBT, Family Intervention
Zhou et al	2018	China	20	895	Anxiety	Psychosocial	CBT
Zhou et al	2018	China	47	1792	Anxiety	Psychosocial	CBT
Zhou et al	2018	China	16	613	Anxiety	Psychosocial	Family Intervention (CBT)
Zhou et al	2018	China	7	357	Anxiety	Psychosocial	Family Intervention (CBT)
Zhou et al	2018	China	7	280	Anxiety	Psychosocial	Family Intervention (CBT)
Zhou et al	2018	China	53	1584	Anxiety	Psychosocial	Family Intervention (CBT)
Zhou et al	2018	China	12	702	Anxiety	Psychosocial	Bibliotherapy (CBT)
Zhou et al	2018	China	47	1599	Anxiety	Psychosocial	Bibliotherapy (CBT)
Zhou et al	2018	China	16	748	Anxiety	Psychosocial	Bibliotherapy (CBT)
Zhou et al	2018	China	7	492	Anxiety	Psychosocial	Bibliotherapy (CBT)
Zhou et al	2018	China	6	415	Anxiety	Psychosocial	Bibliotherapy (CBT)
Zhou et al	2018	China	53	1719	Anxiety	Psychosocial	Bibliotherapy (CBT)
Zhou et al	2018	China	17	1088	Anxiety	Psychosocial	CBT (group), Family Intervention
Zhou et al	2018	China	47	1985	Anxiety	Psychosocial	CBT (group), Family Intervention
Zhou et al	2018	China	26	1114	Anxiety	Psychosocial	CBT
Zhou et al	2018	China	47	2011	Anxiety	Psychosocial	CBT
Zhou et al	2018	China	11	535	Anxiety	Psychosocial	CBT (Internet)
Zhou et al	2018	China	24	1114	Anxiety	Psychosocial	CBT, Family Intervention
Zhou et al	2018	China	47	2011	Anxiety	Psychosocial	CBT, Family Intervention
Zhou et al	2018	China	11	458	Anxiety	Psychosocial	CBT (Internet)
Zhou et al	2018	China	53	1762	Anxiety	Psychosocial	CBT (Internet)
*Zhou et al	2025	China	21	2379	Depression	Physical	Exercise
*Zhou et al	2025	China	14	1586	Anxiety	Physical	Exercise
*Zhou et al	2025	China	19	2152	Depression	Physical	Exercise
*Zhou et al	2025	China	6	680	Anxiety	Physical	Exercise

YoP = Year of Publication. CBT = Cognitive Behavioral Therapy. SG = Second Generation. IPT = Interpersonal Therapy. SUP = Supportive Therapy. FG = First Generation. MBI = Mindfulness-Based Interventions. ABM = Attention Bias Modification. BITS = Behavioral Intervention Technologies. F2F = Face-to-Face. CBM = Cognitive Bias Modification. BT = Behavioral Therapy. BA = Behavioural Activation. * = Studies from Updated Search

### Anxiety

**Fig. 2:**
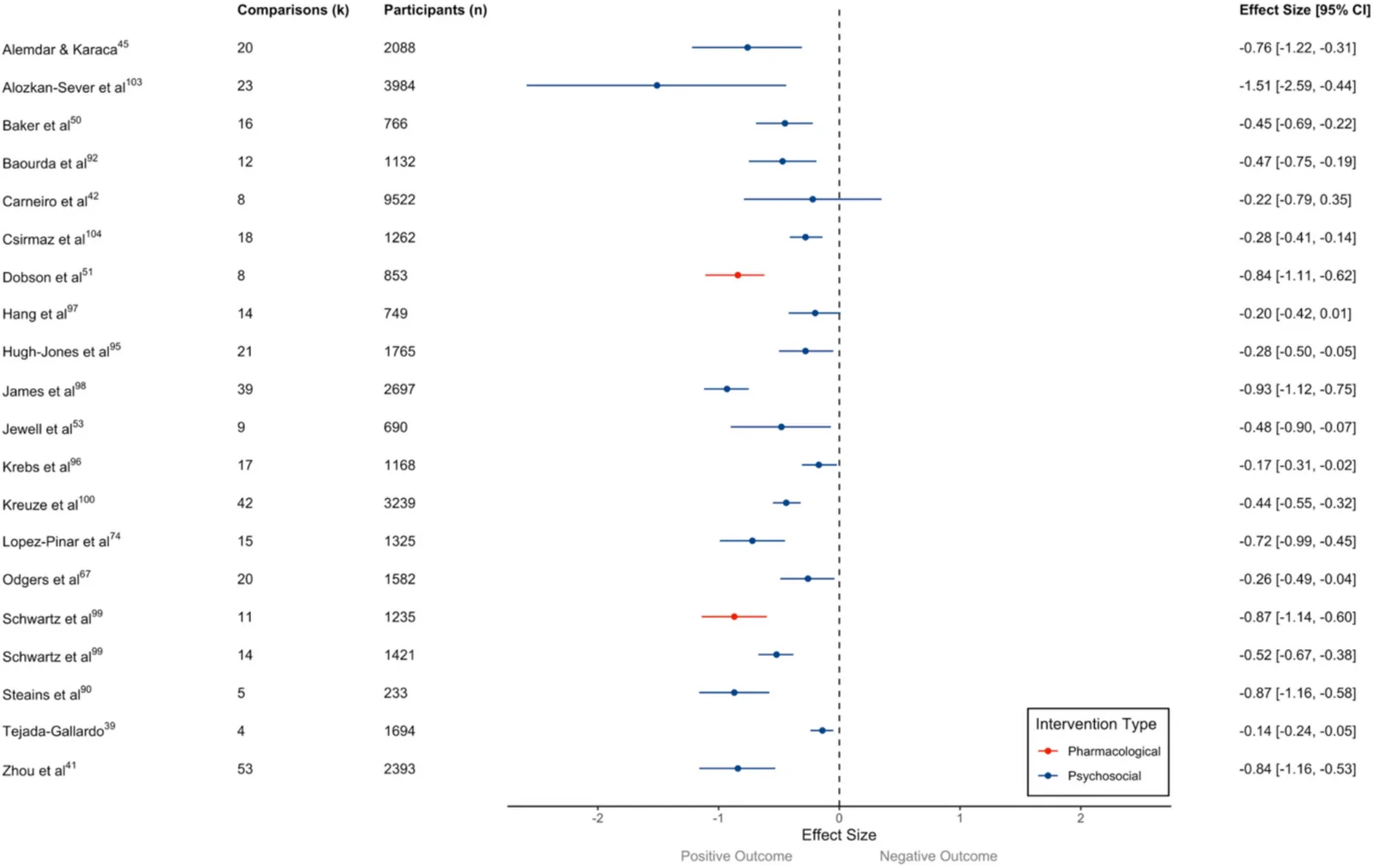
Anxiety forest plot for included meta-analyses. Note. CI = Confidence Interval

For anxiety, there were 230 effects, with *d*s ranging from −2.51 to 0.58 for psychosocial interventions (222 ES) and from −2.87 to −0.06 for pharmacological interventions (6 ES; See Fig. 2). Negative effect sizes indicate symptom reduction and/or increased remission rates compared to controls. There were only two effects for physical interventions for anxiety; therefore, they did not meet the criteria for inclusion in statistical analyses. Conditions included unspecified anxiety disorders (179), unspecified anxious symptoms (44), unspecified depressive symptoms associated with anxiety disorders (5), and selective mutism (2).

Interventions for anxiety were categorized at three levels, by psychosocial versus pharmacological at the least granular (Level 1) and by specific intervention at the most (Level 3). Mid-level categorization (Level 2) separated cognitive and behavioral therapies (acceptance and commitment therapy (ACT), behavioral therapy, bias modification, CBT, exposure therapies, mixed psychosocial therapies including CBT, and family interventions, which were primarily CBT-based) from other psychosocial therapies (Table 2).

**Table 2:** Anxiety levels of operationalization with effect size counts

Total (230)	Level 1	Level 2	Level 3
	Psychosocial (222)	Cognitive & Behavioral Therapies (208)	ACT (4)
			Behavioral Therapy (3) *Not included as ES* < *4*
			Bias Modification (11)
			CBT (144)
			Mixed Psychosocial Therapies including CBT (23)
			Family Intervention (23)
		Other Psychosocial Therapies (14)	Mindfulness-Based Interventions (12)
			Positive Psychology (1) *Not included as ES* < *4*
			Other Psychotherapy (1) *Not included as ES* < *4*
	Pharmacological (6)	Antidepressants (6)	Second-Generation Antidepressants (5)
			First Generation Antidepressants (1) *Not included as ES* < *4*
	Physical (2) *Not included as ES* < *4*	Physical Exercise (2) *Not included as ES* < *4*	Physical Exercise (2) *Not included as ES* < *4*

CBT = Cognitive Behavioral Therapy. ES = Effect sizes.

**Fig. 3:**
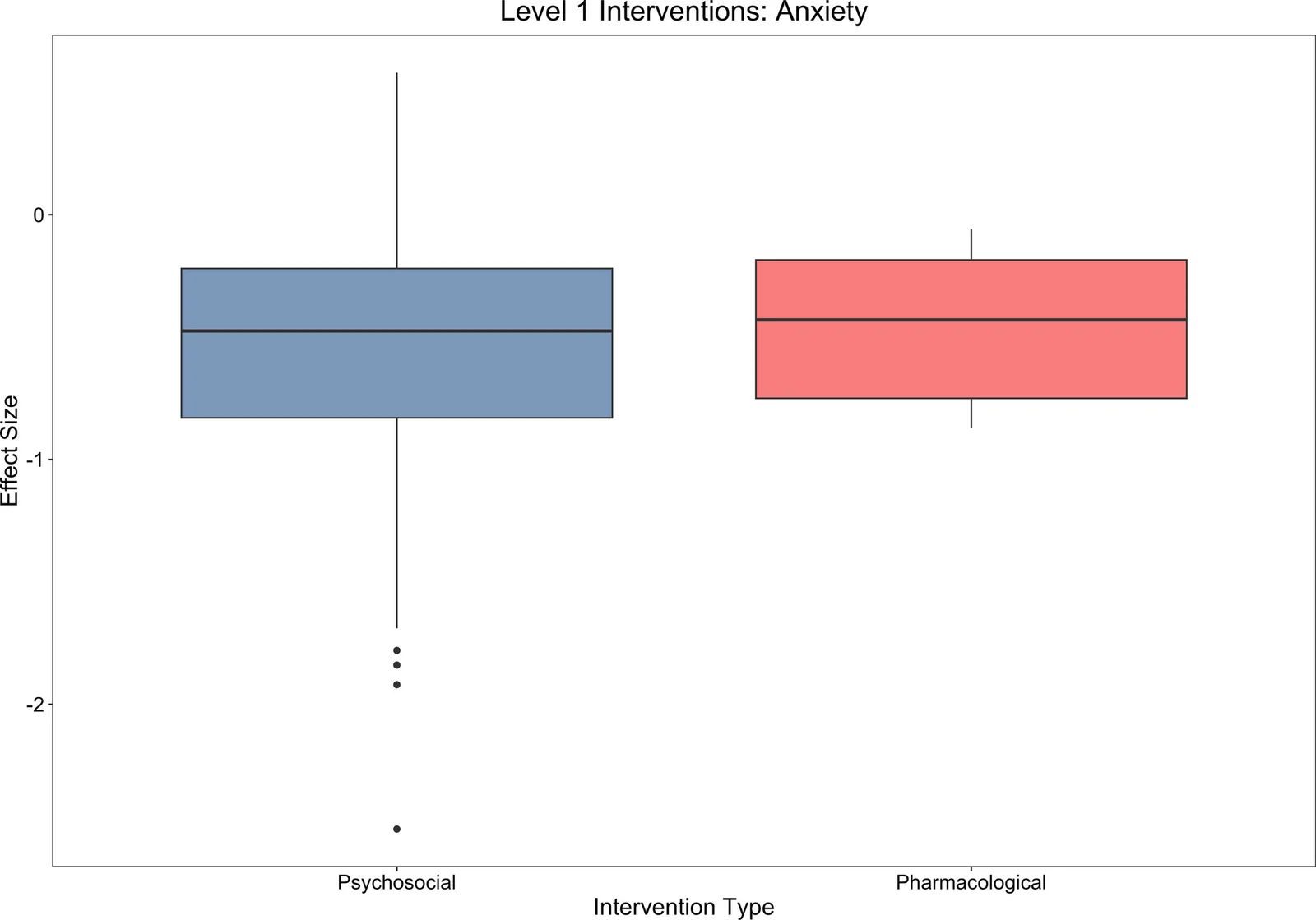
Effectiveness of Level 1 (highest level categorization) interventions for anxiety in children and young people. Note. Negative numbers associated with greater symptom improvement/increased remission rates

For CYP with elevated levels of anxiety, both psychosocial (d = −0.51, 95% CI [−0.56, −0.46], p < 0.001) and pharmacological (d = −0.67, 95% CI [−0.87, −0.46], p < 0.001) interventions significantly improved outcomes compared to controls (Level 1). Effects of the intervention types were not significantly different (t(5.50) = 0.64, p = 0.546) (See Fig. 3).

The following interventions (with > 3 ES) showed significant improvement in anxiety outcomes compared to controls (Level 3): ACT (d = −0.37, 95% CI [−0.69, −0.05], p = 0.02); bias modification (d = −0.21, 95% CI [−0.28, −0.14], p < 0.001); CBT (d = −0.57, 95% CI [−0.63, −0.51], p < 0.001); family interventions (d = −0.52, 95% CI [−0.72, −0.32], p < 0.001); mindfulness-based interventions (MBIs) (d = −0.21, 95% CI [−0.29, −0.13], p < 0.001); mixed psychosocial therapies including CBT (d = −0.39, 95% CI [−0.49, −0.28], p < 0.001); and second-generation antidepressants (d = −0.71, 95% CI [−0.93, −0.48], p < 0.001).

**Fig. 4:**
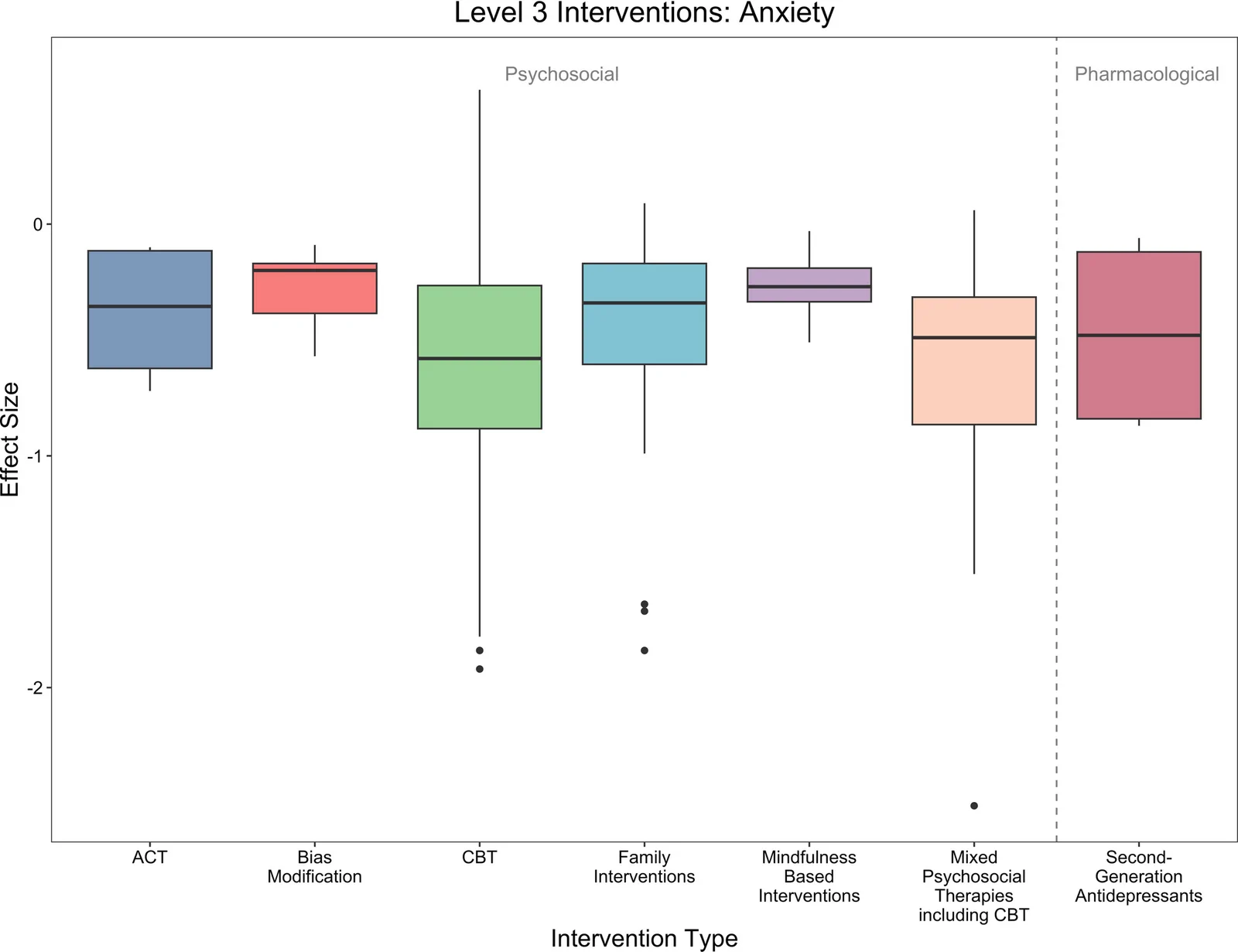
Effectiveness of Level 3 (most granular categorization) interventions for anxiety in children and young people. Note. CBT = Cognitive Behavioral Therapy. w/= with. Negative numbers associated with greater symptom improvement/increased remission rates

Levene’s test indicated unequal variances (F(6,215) = 2.40, p = 0.029), so a Welch’s ANOVA was conducted. There was significant variation in the effects of the intervention types (F(6,21.30) = 7.12, p < 0.001), (Fig. 4). See Table 3 for full results. Note that one effect size was identified as an outlier (± 3 standard deviations from the mean), and when removed, mixed psychosocial therapies including CBT were also significantly more effective than MBIs (d = −0.30 [−0.60, −0.01], p = 0.041). No other anxiety results were substantially changed by the inclusion of outliers.

**Table 3:** Level 3 anxiety pairwise comparison table

	**Acceptance & Commitment Therapy (ACT)**	**Bias Modification**	**Cognitive Behavioral Therapy (CBT)**	**Family Interventions**	**Mindfulness Based Interventions (MBIs)**	**Mixed Psychosocial Therapies including CBT**	**Second-Generation Antidepressants**
Acceptance & Commitment Therapy (ACT)	*NA*	*NA*	*NA*	*NA*	*NA*	*NA*	*NA*
Bias Modification	0.11 [−0.78, 1.00], p = 0.988	*NA*	*NA*	*NA*	*NA*	*NA*	*NA*
Cognitive Behavioral Therapy (CBT)	−0.21 [−1.11, 0.70], p = 0.844	−0.32 [−0.51, −0.12], p < 0.001***	*NA*	*NA*	*NA*	*NA*	*NA*
Family Interventions	−0.13 [−0.92, 0.66], p = 0.990	−0.24 [−0.63, 0.15], p = 0.452	0.07 [−0.31, 0.45], p = 0.996	*NA*	*NA*	*NA*	*NA*
Mindfulness Based Interventions (MBIs)	0.12 [−0.79, 1.03], p = 0.980	0.01 [−0.19, 0.21], p = 1.000	0.32 [0.16, 0.49], p < 0.001***	0.25 [−0.13, 0.63], p = 0.373	*NA*	*NA*	*NA*
Mixed Psychosocial Therapies including CBT	−0.27 [−1.06, 0.52], p = 0.812	−0.38 [−0.78, 0.02], p = 0.077	−0.06 [−0.45, 0.33], p = 0.999	−0.13 [−0.64, 0.37], p = 0.982	−0.39 (−0.78, 0.01], p = 0.055	*NA*	*NA*
Second-Generation Antidepressants	−0.09 [−1.02, 0.84], p = 0.999	−0.20 [−1.02, 0.62], p = 0.892	0.11 [−0.72, 0.95], p = 0.990	0.04 [−0.74, 0.83], p = 1.000	−0.21 [−1.04, 0.62], p = 0.865	0.18 [−0.61, 0.96], p = 0.972	*NA*

Note: Comparisons between interventions where ES > 3

Reported as: MD [lower 95% confidence interval, upper 95% confidence interval], p-value.

Significance noted as p-values: < 0.05 (*), < 0.01 (**), < 0.001 (***).

**Fig. 5:**
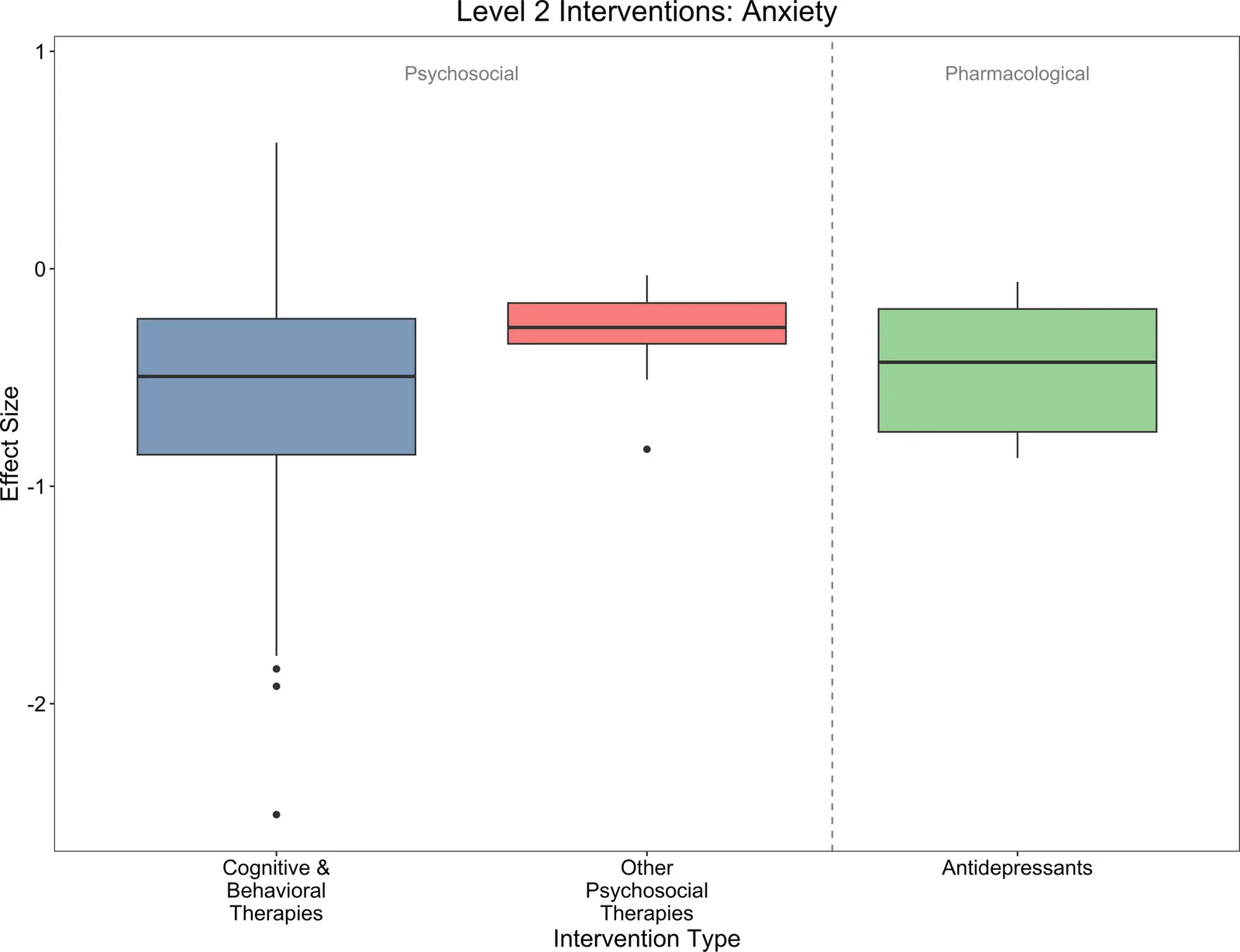
Effectiveness of Level 2 (mid-level categorization) interventions for anxiety in children and young people. Note. CBT = Cognitive Behavioral Therapy. Negative numbers associated with greater symptom improvement/increased remission rates

For grouped interventions (Level 2), Levene’s test again indicated unequal variances (F(2,225) = 3.96, p = 0.020). A Welch’s ANOVA revealed significant variation in the effects of the intervention types (F(2,11.7) = 9.15, p = 0.004). Games-Howell post-hoc tests showed that cognitive and behavioral therapies were significantly more effective than other psychosocial therapies (MD = −0.27, 95% CI [−0.43, −0.12], p < 0.001). Antidepressants were not significantly different from either psychosocial category (See Table 4 and Fig. 5).

**Table 4:** Level 2 anxiety pairwise comparison table

	**Antidepressants**	**CBT-Based Interventions**	**Other Psychosocial Therapies**
**Antidepressants**	*NA*	*NA*	*NA*
**Cognitive & Behavioral Therapies**	−0.11 [−0.56, 0.34], p = 0.739	*NA*	*NA*
**Other Psychosocial Therapies**	0.16 [−0.29, 0.62], p = 0.552	0.27 [0.12, 0.43], p < 0.001***	*NA*

CBT = Cognitive Behavioral Therapy

Reported as: MD [lower 95% confidence interval, upper 95% confidence interval], p-value.

Significance noted as p-values: < 0.05 (*), < 0.01 (**), < 0.001 (***).

### Depression

**Fig. 6:**
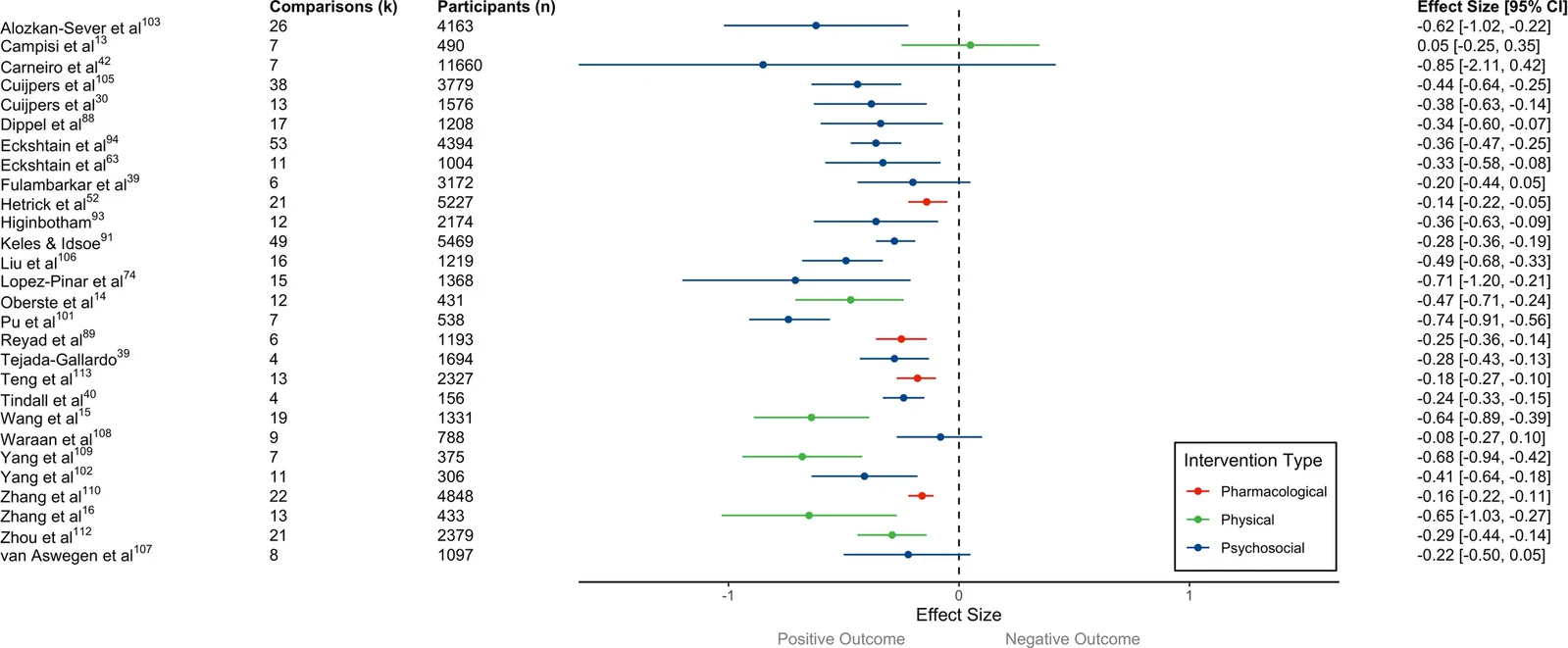
Depression forest plot for included meta-analyses. Note. CI = Confidence Interval

For depression there were 161 effects (See Fig. 6), with *d*s ranging from −1.14 to 0.06 for psychosocial interventions (108 ES), −0.43 to −0.03 for pharmacological interventions (22 ES), and −1.30 to 0.05 for physical interventions (31 ES). Conditions included unspecified depressive symptoms (86), unspecified depressive disorders (55), major depressive disorder (22), and unspecified anxious symptoms associated with depressive disorders (1).

Interventions for depression were also categorized at three levels. Alongside psychosocial and pharmacological interventions, physical interventions were included in depression Level 1. At Level 2, psychosocial interventions were separated into cognitive and interpersonal therapies (CBT, family interventions, interpersonal therapy (IPT), and mixed cognitive and interpersonal therapies) and other psychosocial therapies. Pharmacological interventions were separated into mixed antidepressants and second-generation antidepressants, and exercise interventions were again included. Level 3 included various psychosocial interventions alongside the two pharmacological categories and exercise interventions (See Table 5).

**Table 5:** Depression levels of operationalization with effect size counts

Total (161)	Level 1	Level 2	Level 3
	Psychosocial (98) *Total 108, 10 ES excluded only at this level due to study overlap*	Cognitive and Interpersonal Therapies (104)	ACT (4)
			CBT (27)
			Family Intervention (11)
			Mixed Cognitive and Interpersonal Therapies (55)
			Interpersonal Therapy (7)
		Other Psychosocial Therapies (4)	MBIs (3) *Not included as ES* < *4*
			Positive Psychology (1) *Not included as ES* < *4*
	Pharmacological (22)	Second-Generation Antidepressants (12)	Second-Generation Antidepressants (12)
		Mixed Antidepressants (10)	Mixed Antidepressants (10)
	Physical (31)	Physical Exercise (31)	Physical Exercise (31)

CBT = Cognitive Behavioral Therapy. ES = Effect sizes

Note that of the 10 psychosocial effects came from meta-analyses that exceeded the threshold of 75% primary study overlap with another included meta-analysis at Level 1, but were part of different sub-categories at Levels 2 and 3 due to increased specificity of certain meta-analyses. Therefore, these 10 effects were excluded at Level 1 but included at Levels 2 and 3

**Fig. 7:**
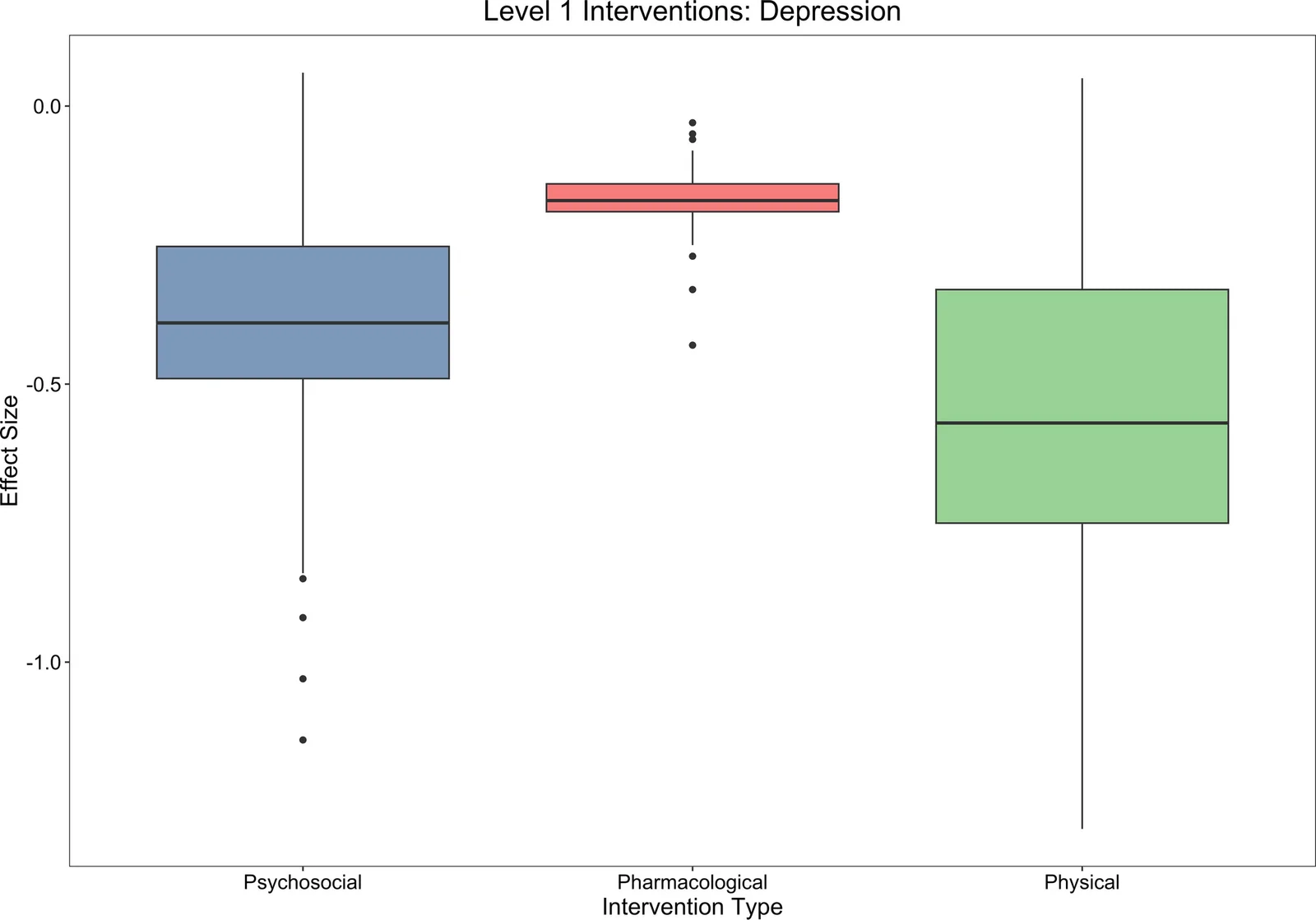
Effectiveness of Level 1 (highest level categorization) interventions for depression in children and young people. Note. Negative numbers associated with greater symptom improvement/increased remission rates

For CYP with elevated levels of depression, psychosocial (d = −0.35, CI [−0.39, −0.32], p < 0.001), pharmacological (d = −0.17, CI [−0.20, −0.15], p < 0.001), and physical (d = −0.49, CI [−0.57, −0.40], p < 0.001) interventions all significantly improved outcomes compared to controls (Level 1). Levene’s test indicated heterogeneity of variances (F(2,151) = 11.44, p < 0.001). A Welch’s ANOVA revealed a significant effect of intervention type on depression outcomes (F(2,65.3) = 50.1, p < 0.001). Games-Howell post-hoc tests indicated that physical (MD = −0.42, CI [−0.57, −0.28], p < 0.001) and psychosocial (MD = −0.24, CI [−0.31, −0.17], p < 0.001) interventions were both associated with more symptom improvement and higher remission rates than pharmacological interventions (Fig. 7). Physical interventions were also associated with better outcomes than psychosocial (MD = −0.18, CI [−0.34, −0.03], p = 0.015).

The following interventions (with > 3 ES) showed significant improvement in depression outcomes compared to controls (Level 3): ACT (d = −0.51, CI [−0.86, −0.15], p = 0.005); CBT (d = −0.28, CI [−0.33, −0.23], p < 0.001); family interventions (d = −0.24, CI [−0.33, −0.15], p < 0.001); interpersonal therapy (d = −0.71, CI [−0.88, −0.54], p < 0.001); mixed antidepressants (d = −0.17, CI [−0.21, −0.13], p < 0.001); mixed cognitive and interpersonal therapies (d = −0.35, CI [−0.38, −0.31], p < 0.001); physical exercise (d = −0.49, CI [−0.57, −0.40], p < 0.001); and second-generation antidepressants (d = −0.18, CI [−0.22, −0.14], p < 0.001).

**Fig. 8:**
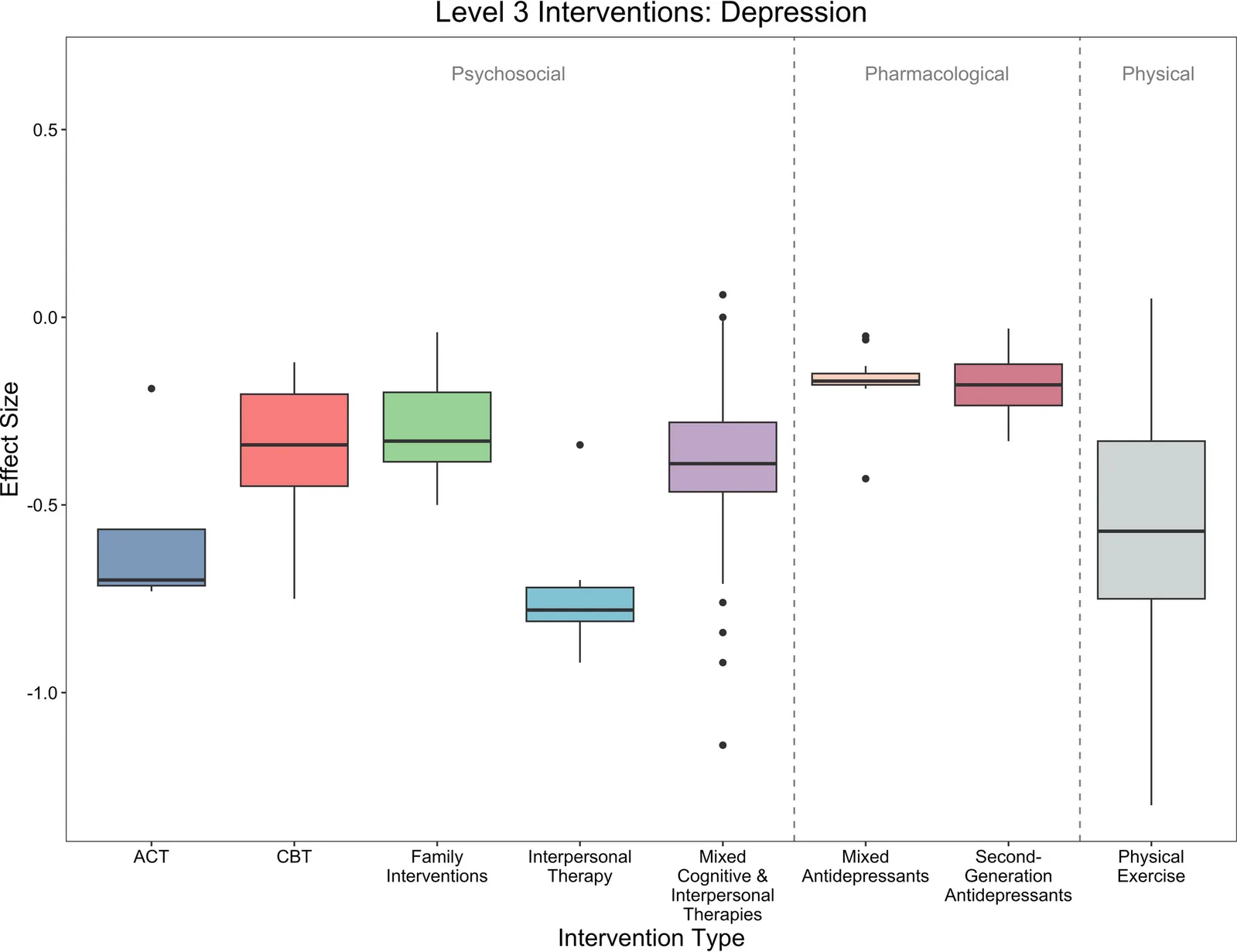
Effectiveness of Level 3 (most granular categorization) interventions for depression in children and young people. Note. CBT = Cognitive Behavioral Therapy. w/= with. Negative numbers associated with greater symptom improvement/increased remission rates

At Level 3, Levene’s test indicated heterogeneity of variance (F(9, 154) = 3.35, p < 0.001), so a Welch’s ANOVA was conducted which revealed significant variation in the effects of the intervention types (F(7, 28.4) = 16.6, p < 0.001) (See Fig. 8). Notably, IPT and exercise interventions did not significantly differ (MD = −0.13, CI [−0.45, 0.19], p = 0.831), but were both associated with significantly greater improvements than most other interventions. CBT and mixed cognitive and interpersonal therapies were also both associated with greater improvement than mixed or second-generation antidepressants. See Table 6 for full results.

**Table 6:** Level 3 depression pairwise comparison table

	**Acceptance & Commitment Therapy (ACT)**	**Mixed Antidepressants**	**Cognitive Behavioral Therapy (CBT)**	**Exercise**	**Family Interventions**	**Interpersonal Therapy (IPT)**	**Mixed Cognitive & Interpersonal Therapies**	**Second-Generation Antidepressants**
**Acceptance & Commitment Therapy (ACT)**	*NA*	*NA*	*NA*	*NA*	*NA*	*NA*	*NA*	*NA*
**Mixed Antidepressants**	0.41 [−0.38, 1.20], p = 0.268	*NA*	*NA*	*NA*	*NA*	*NA*	*NA*	*NA*
**Cognitive Behavioral Therapy (CBT)**	0.24 [−0.54, 1.02], p = 0.660	−0.17 [−0.30, −0.04], p = 0.003**	*NA*	*NA*	*NA*	*NA*	*NA*	*NA*
**Exercise**	−0.02 [−0.73, 0.69], p = 1.000	−0.43 [−0.63, −0.23], p < 0.001***	−0.26 [−0.47, −0.05], p = 0.006**	*NA*	*NA*	*NA*	*NA*	*NA*
**Family Interventions**	0.29 [−0.45, 1.04], p = 0.522	−0.12 [−0.30, 0.06], p = 0.352	0.05 [−0.13, 0.24], p = 0.975	0.31 [0.07, 0.55], p = 0.003**	*NA*	*NA*	*NA*	*NA*
**Interpersonal Therapy (IPT)**	−0.15 [−0.85, 0.55], p = 0.953	−0.56 [−0.86, −0.26], p = 0.001**	−0.39 [−0.69, −0.09], p = 0.011*	−0.13 [−0.45, 0.19], p = 0.831	−0.44 [−0.75, −0.13], p = 0.004**	*NA*	*NA*	*NA*
**Mixed Cognitive & Interpersonal Therapies**	0.18 [−0.60, 0.96], p = 0.830	−0.23 [−0.35, −0.11], p < 0.001***	−0.06 [−0.19, 0.07], p = 0.875	0.20 [0.00, 0.41], p = 0.057	−0.11 [−0.29, 0.07], p = 0.483	0.33 [0.04, 0.63], p = 0.028*	*NA*	*NA*
**Second-Generation Antidepressants**	0.40 [−0.39, 1.19], p = 0.281	−0.01 [−0.13, 0.11], p = 1.000	0.16 [0.03, 0.29], p = 0.005**	0.42 [0.22, 0.62], p < 0.001***	0.11 [−0.07, 0.29], p = 0.446	0.55 [0.25, 0.85], p = 0.001**	0.22 [0.10, 0.34], p < 0.001***	*NA*

Comparisons between interventions where ES > 3

Reported as: MD [lower 95% confidence interval, upper 95% confidence interval], p-value.

Significance noted as p-values: < 0.05 (*), < 0.01 (**), < 0.001 (***).

**Fig. 9:**
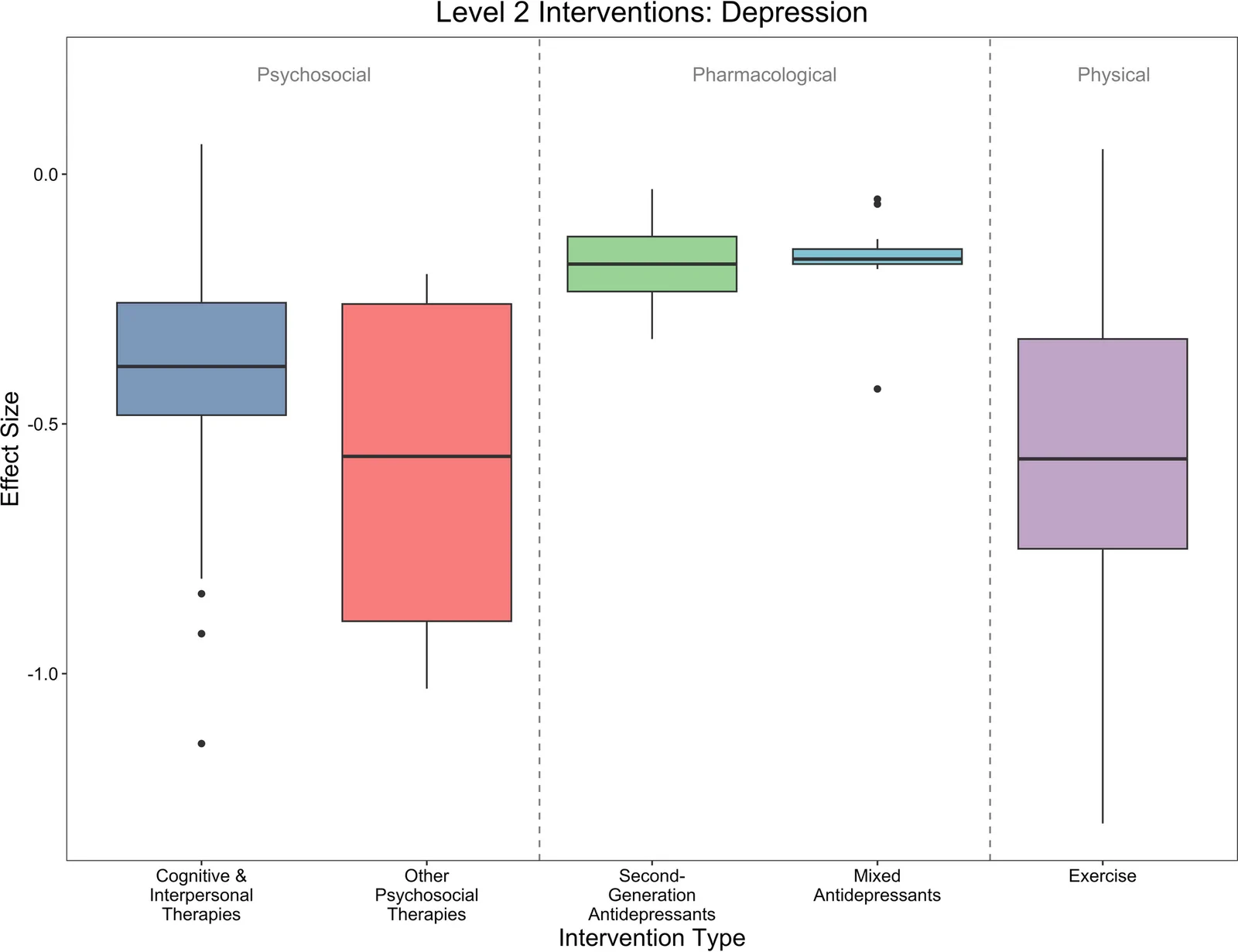
Effectiveness of Level 2 (mid-level categorization) interventions for depression in children and young people. Note: CBT = Cognitive Behavioral Therapy. Negative numbers associated with greater symptom improvement/increased remission rates

When grouped (Level 2), Levene’s test indicated unequal variance (F(4,159) = 7.91, p < 0.001). A Welch’s ANOVA revealed significant variation in the effects of the intervention types (F(4,17.6) = 22.5, p < 0.001), (See Fig. 9). Exercise was associated with significantly more improvements than cognitive and interpersonal therapies (MD = −0.20, CI [−0.38, −0.02], p = 0.021), and both were associated with significantly more improvements than mixed or second-generation antidepressants. Two effect sizes were identified as outliers (± 3 standard deviations from the mean), and when removed there was no longer a significant difference between exercise and cognitive and interpersonal interventions. No other results were significantly changed by the inclusion of outliers. There was a small sample size of other psychosocial therapies (4), and no significant differences with other interventions emerged. See Table 7 for full results.

**Table 7:** Level 2 depression pairwise comparison table

	**Cognitive & Interpersonal Therapies**	**Exercise Interventions**	**Mixed Antidepressants**	**Other Psychosocial Therapies**	**Second-Generation Antidepressants**
Cognitive & Interpersonal Therapies	*NA*	*NA*	*NA*	*NA*	*NA*
Exercise Interventions	−0.20 [−0.38, −0.02], p = 0.021*	*NA*	*NA*	*NA*	*NA*
Mixed Antidepressants	0.23 [0.14, 0.32], p < 0.001***	0.43 [0.25, 0.61], p < 0.001***	*NA*	*NA*	*NA*
Other Psychosocial Therapies	−0.19 [−1.27, 0.89], p = 0.875	0.01 [−1.02 1.04], p = 1.000	−0.42 [−1.50, −0.66], p = 0.418	*NA*	*NA*
Second-Generation Antidepressants	0.22 [0.13, 0.32], p < 0.001***	0.42 [0.24, 0.60], p < 0.001***	−0.01 [−0.11, 0.10], p = 0.999	0.41 [−0.67, 1.49], p = 0.433	*NA*

CBT = Cognitive Behavioral Therapy

Reported as: MD [lower 95% confidence interval, upper 95% confidence interval], p-value.

Significance noted as p-values: < 0.05 (*), < 0.01 (**), < 0.001 (***).

### Anxiety vs depression

There were also some significant differences in the effectiveness of the same interventions for anxiety versus depression. Comparisons were conducted where categorizations included roughly equivalent therapies and both anxiety and depression had at least 4 outcomes. This included: at Level 1, psychosocial and pharmacological interventions; at Level 2, other psychosocial therapies (primarily including MBIs and positive psychology); at Level 3, ACT, CBT, family interventions, and second-generation antidepressants.

Psychosocial interventions at Level 1 (MD = −0.14, CI [−0.21, −0.06], t(314.30) = −3.45, p < 0.001) and CBT at Level 3 (MD = −0.23, CI [−0.34, −0.15], t(118.84) = −5.05, p < 0.001) were both associated with significantly higher levels of improvement for anxiety than for depression.

There was no discernible difference between the remaining interventions for anxiety versus depression, including for pharmacological interventions at Level 1 (MD = −0.28, CI [−0.65, −0.08], t(5.15) = −2.00, p = 0.100), other psychosocial therapies at Level 2 (MD = 0.30, CI [−0.34, 0.93], t(3.41) = 1.39, p = 0.248), and ACT (MD = 0.20, CI [−0.31, 0.71], t(5.77) = 0.96, p = 0.376), family interventions (MD = −0.23, CI [−0.48, 0.02], t(27.95) = −1.86, p = 0.073), and second-generation antidepressants at Level 3 (MD = −0.29, CI [−0.77, 0.18], t(4.17) = −1.70, p = 0.161).

Two outliers (± 3 standard deviations from the mean) were identified between anxiety and depression outcomes, but their inclusion did not substantially impact results.

**Fig. 10:**
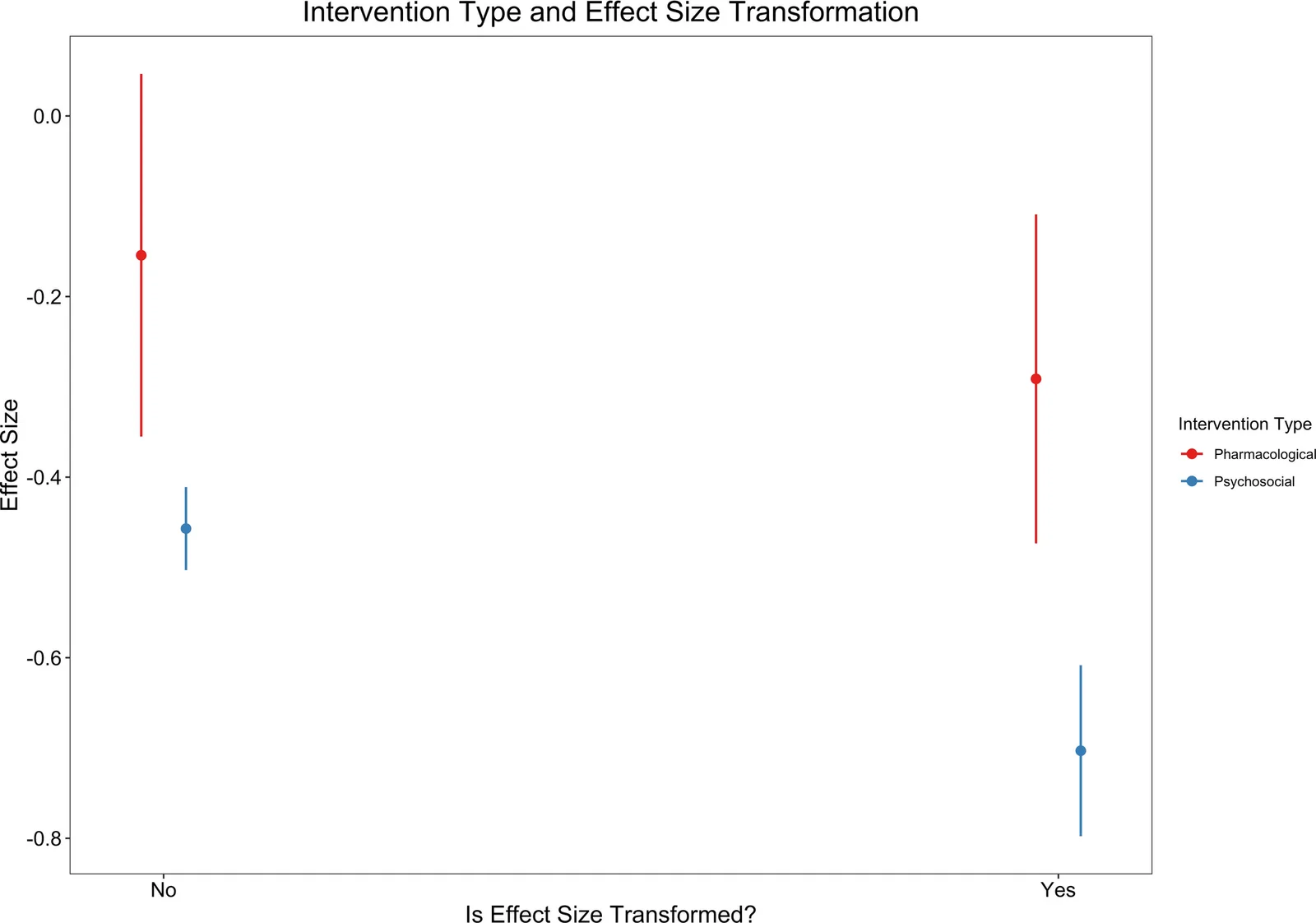
Sensitivity Analysis: Interaction effect of effect size transformation with pharmacological versus psychosocial intervention effectiveness for anxiety and depression combined. *Note. Negative numbers associated with greater symptom improvement/increased remission rates*

To examine the robustness of findings, a sensitivity analysis was conducted comparing the results of untransformed and transformed (converted to Cohen’s d) effect sizes. There was no significant interaction between intervention type and effect size transformation (F(1,357) = 0.54, p = 0.461), indicating that the difference between pharmacological and psychosocial interventions did not significantly vary depending on whether effect sizes were transformed or not. Regardless of transformation, psychosocial interventions were associated with significantly more improvement than pharmacological interventions overall (F(1,359) = 13.87, p < 0.001), and transformed effect sizes represented significantly less improvement than untransformed effect sizes (F(1,359) = 12.12, p < 0.001) (See Fig. 10). Therefore, it can be assumed that the transformation of effect sizes did not significantly inflate the apparent effectiveness of interventions in the results.

**Fig. 11:**
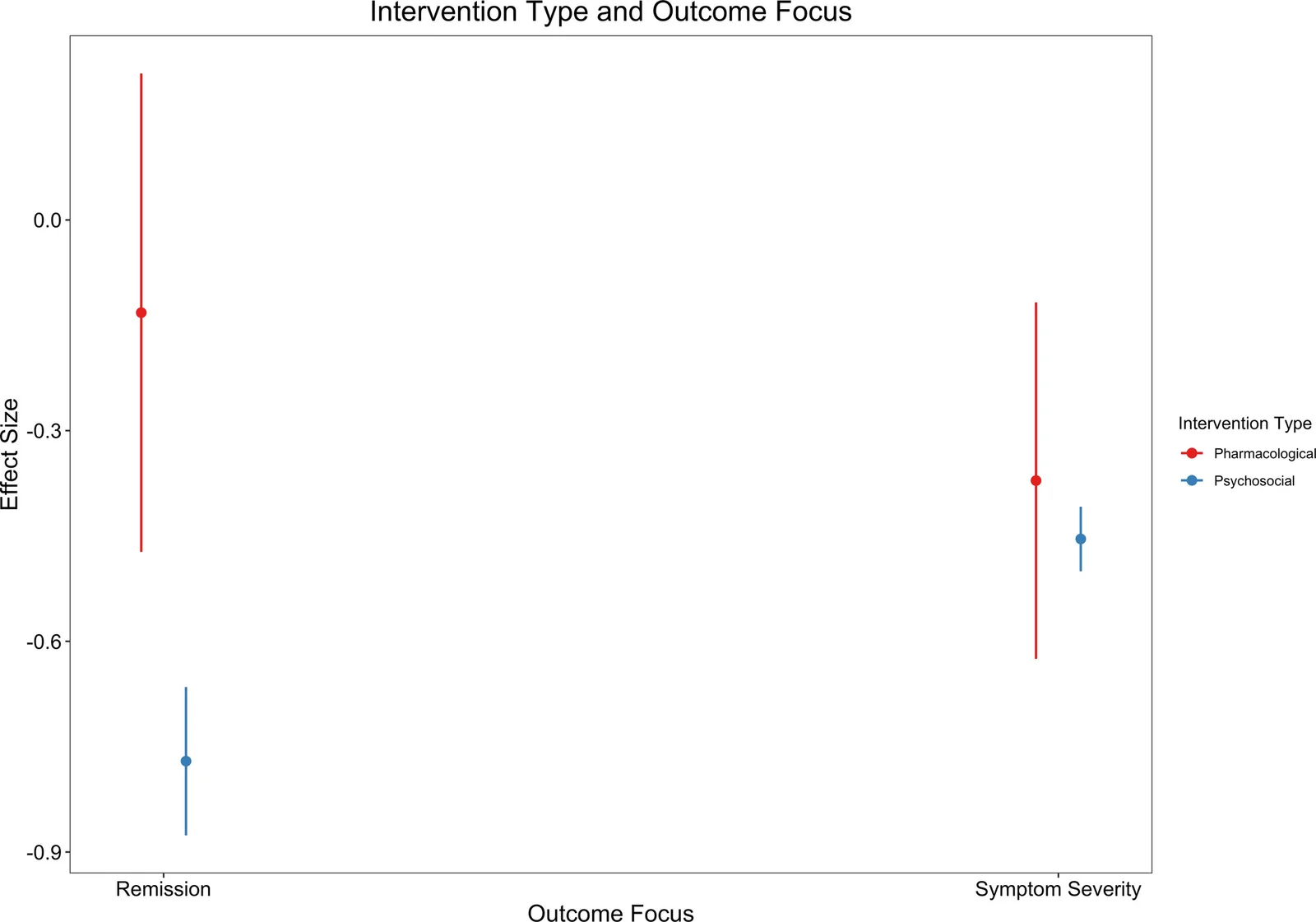
Moderation: Interaction effect of outcome focus with pharmacological versus psychosocial intervention effectiveness for anxiety and depression combined. Note. Negative numbers associated with greater symptom improvement/increased remission rates

Moderation analyses were also conducted via two-way ANOVA where possible. Psychosocial interventions were significantly more effective than pharmacological interventions in targeting remission overall for CYP with depression or anxiety but there was no significant difference in the effectiveness of the two intervention types for reducing symptom severity (interaction: F(1,337) = 6.16, p = 0.014) (See Fig. 11).

There was not enough data to calculate this interaction effect for physical interventions, or to check for moderation of anxiety and depression outcomes separately. Sample context (clinical, community), outcome reporter (parent, clinician, child, other), and specific diagnosis (run separately for anxiety and depression) did not significantly moderate outcomes. However, though the interaction between specific diagnosis for depression (depressive disorder versus depressive symptoms) and intervention type (only enough data for psychosocial versus physical) was not significant (F(1,134) = 1.95, p = 0.165), and therefore did not moderate the relationship, it is notable that a significant main effect of was identified (F(1,136) = 13.85, p < 0.001), where physical interventions were associated with significantly more improvement than psychosocial interventions for depressive symptoms alone.

## Discussion

This umbrella review details quantitative evidence from 43 meta-analyses and 391 effect sizes exploring the effectiveness of psychological, pharmacological, and physical interventions as standalone treatments for CYP with anxiety or depression. Analyses revealed significant differences in the effectiveness of interventions, particularly for depression, and a significant discrepancy in the effectiveness of the same interventions when treating anxiety versus depression.

For CYP with anxiety, psychosocial and pharmacological interventions appeared equally effective in the included evidence base. However, more research was available on psychosocial interventions as standalone treatments than pharmacological interventions, likely aligning with current policy recommendations that suggest that pharmacological treatments should be introduced only when standalone psychosocial interventions have had limited effect, and then used in combination with other treatments [20]. ACT, bias modification, CBT, family interventions, MBIs, second-generation antidepressants, and mixed psychosocial therapies including CBT all reduced symptoms and increased rates of remission.

Differences emerged when psychosocial treatments were separated into cognitive and behavioral therapies and other psychosocial therapies. Cognitive and behavioral therapies were associated with greater improvement than other psychosocial therapies, such as MBIs and positive psychology, and were similarly effective to antidepressants. Current public health recommendations for CYP with anxiety highlight cognitive and behavioral therapies as an effective first-line treatment [20, 21]. Clearer guidance is needed on antidepressants, as these findings demonstrate that they could be similarly effective for short-term treatment of anxiety. However, research beyond the scope of this review suggests that antidepressant effects may not sustain as well over time as those from psychosocial therapies [64, 65]. This, alongside the potential risks of physical and mental side effects in the short and long term [66], may be why antidepressants are not often recommended as a standalone treatment for anxiety despite their apparent effectiveness.

Additionally, CBT specifically was associated with significantly better outcomes than MBIs, as were mixed psychosocial therapies including CBT when a single outlier was removed. While this aligns with the literature [43, 58, 67], policy recommendations for treating anxiety in CYP still support the use of MBIs, particularly in schools [68, 69]. Compared to other psychosocial interventions such as CBT, which focus on teaching cognitive and behavioral strategies to manage anxious symptoms overtime [4, 9], MBIs may be less effective because they equip CYP with fewer proactive coping mechanisms for addressing symptoms in the long-term [58, 67], or because they are often facilitated by non-mental health professionals, such as school or charity staff [43]. CBT also appeared significantly more effective than bias modification. This supports literature in the field suggesting that bias modification, despite being cost-effective, may have limited clinical effects compared to other standalone treatments [70] and may be more effective when used in combination with CBT due to its narrower therapeutic focus [55].

For CYP with depression, psychosocial, pharmacological, and physical interventions all appeared effective. Physical interventions were associated with the greatest improvements, followed by psychosocial interventions, then by pharmacological interventions, which appeared comparatively least effective. Of the specific interventions, IPT, ACT, and physical exercise were associated with the greatest improvements. Interpretation of these differences requires caution. Physical interventions are most commonly used to treat mild and subthreshold depressive symptoms, which are generally more responsive to treatment than moderate and severe depression [29]. In contrast, pharmacological interventions are more often used for moderate to severe cases [28], which may partially explain their comparatively smaller effects. Within pharmacological approaches in this evidence base, there was no significant difference between second-generation and mixed antidepressants. For psychosocial interventions, which are used to treat depression with a wider range of severity, cognitive and interpersonal therapies appeared more effective than second-generation or mixed antidepressants. However, the only variation in effectiveness between specific therapies came from IPT. Although ACT also appeared highly effective, the number of effect sizes in this review was limited, and outcomes did not differ significantly from those of other therapies.

The differences in intervention effectiveness identified appear closely linked to variation in depression severity within the evidence base. While differences between intervention types were less clear for diagnosed depressive disorders, physical interventions were significantly more effective than psychosocial interventions for subthreshold symptoms. This pattern is reflected in the distribution of effect sizes: approximately 80% of physical intervention effects were derived from subthreshold symptoms, compared to 66% for psychosocial interventions and none for pharmacological interventions.

There has been considerable debate in the literature regarding the comparative effectiveness of psychosocial therapy and pharmacology for treating depression [71, 72]. This debate is compounded by inconsistent policy recommendations for CYP [12, 27, 28]. The distinctions evidenced by this review are significant emerging findings, demonstrating that, in the included evidence base, antidepressants may be less effective for CYP than other interventions, despite being developed to treat depression [73]. As policy recommendations suggest [28], antidepressants may still be an effective treatment standalone or in combination for CYP with moderate to severe depression, especially where symptoms are enduring and other therapeutic methods have been ineffective, but this is beyond the scope of this review. Physical interventions are typically not highlighted as a first-line treatment in practice recommendations [12, 27] or in the intervention debate [71, 72]. Given the promising findings of this review, additional research on physical interventions, particularly for CYP with mild depression, may help guide future discussions and recommendations if results are replicable and feasible in clinical practice settings and when directly compared to alternative treatments. Contrastingly, IPT is often included in policy and practice as an alternative psychosocial treatment to CBT, a recommendation supported by these findings [12, 27]. Despite appearing more effective, however, IPT is considerably under-researched compared to CBT (with seven IPT effect sizes in this depression review, and 27 CBT). This review adds to existing calls for additional evidence on IPT [12], and highlights the need for further research into the mechanisms that may underlie its effectiveness for depression relative to other treatments. Similarly, though ACT showed promise for the treatment of depression in CYP in this review, comparisons to other interventions were non-significant, and research was limited and recent – with four effect sizes from a single 2025 review [74] – highlighting the need for additional meta-analytic research.

In comparisons of the same interventions for anxiety versus depression, effectiveness appeared either similar or in favor of anxiety. There were no significant differences in the effectiveness of pharmacological interventions, including antidepressants, for treating anxiety versus depression. This evidence aligns with psychiatric initiatives that encourage adopting neuroscience-based nomenclature for psychotropics, rather than naming them according to one specific diagnosis that they may or may not best treat [75]. Psychosocial interventions and CBT were both associated with better outcomes for anxiety than depression. In the context of emerging literature which calls into question the effectiveness of existing interventions for depression generally [76, 77], and considering the potential long-term effects of untreated or undertreated depression in CYP [7], these findings highlight a need for further research into the development of new or modified depression interventions.

There were several interventions that only had sufficient data for one diagnosis and therefore could not be compared. For instance, while IPT appeared to be the most effective psychosocial therapy for the treatment of depression, no reviews explored IPT for anxiety. Some recent research on comorbid anxiety and depression suggests that IPT may effectively reduce anxiety symptoms, but further research is needed [78, 79]. Similarly, exercise, which appeared highly effective in depression trials, had too limited data for anxiety (2 effect sizes) to include in this review’s statistical analysis. Of the two effects that did meet the inclusion criteria, neither indicated that physical activity was more beneficial for anxiety than controls [41]. While meta-analytic research around exercise and anxiety is increasing, and some indicate positive associations, most reviews currently focus on the prevention of anxiety symptoms or the reduction of anxiety in CYP who are healthy or have physical or neurodevelopmental diagnoses [15, 46, 80], rather than those with established above-baseline anxiety related to mental health, as was the focus of this review. Given the effectiveness of other overlapping interventions for anxiety and the emerging evidence, policymakers and practitioners should monitor developments in IPT and exercise for these CYP and consider expanding recommendations for anxiety treatments accordingly.

Our review provides a comprehensive update of recent literature through February 2026, including meta-analyses from four continents. However, it has limitations that should be acknowledged. First, there were some limitations in the available data. Although demographic data including gender and race ratios were extracted, this information was unavailable for 60–100% of effects (See Supplementary S8), and therefore, moderation analyses with this data were not feasible. Missing data also prevented analysis on treatment frequency, duration, and follow-up. Additionally, though we could use diagnosis (subthreshold symptoms vs disorder) as a proxy for baseline severity, actual baseline severity may differ within these categories, and this information was not widely available in reviews. This may be especially meaningful for depression results, where physical interventions appeared most effective but are also most often used to treat subthreshold and mild depression [29]. Some variation in the effectiveness of treatments may be explained by baseline severity, which should be compared directly in future research when possible. Second, effect sizes were standardized to Cohen’s* d* to enable the utilization of higher-level statistical tests. Transformation assumes equal groups. While we attempted to extract intervention and control group sizes, for over half of the included effects this data was unavailable, and most meta-analyses did not control for variation in group size across individual studies. A sensitivity analysis indicated that the transformation of effect sizes did not significantly moderate the relationship between intervention type (psychosocial versus pharmacological) and outcome. However, there was not enough power to assess moderation for anxiety and depression outcomes separately. While transformation of effect sizes aligns with statistical methods in previous umbrella reviews [81–83], it provides important context for interpretation of results, and comparisons were run where possible excluding transformed data. For anxiety, untransformed data indicated that antidepressants may be less effective than cognitive and behavioral therapies (MD = 0.41, CI [0.27, 0.56], p < 0.001) or other psychosocial therapies (MD = 0.20, CI [0.04, 0.37], p = 0.019), where otherwise there were no significant differences. With this context, the impact of antidepressants may be somewhat more modest than indicated in initial results, though notably differences remained non-significant when pharmacological and psychosocial interventions were compared at the highest level. For depression, no significant changes resulted from the removal of transformed data. Third, of the original 569 effect sizes, only 6 reported results exclusively for CYP with co-morbid depression and anxiety, meaning there was not enough data to explore treatments across co-occurring diagnoses. This may limit practice implications, as rates of comorbidity are high – generally estimated at over 50% in the general population [84], and potentially higher in symptom overlap [85]. Relatedly, no included effects reported combined psychosocial and pharmacological interventions, focusing exclusively on standalone treatments. This may also limit practical application, as combination treatment is often recommended with pharmacology [11, 20, 28], and has generally been found to be more effective than either approach individually [86, 87]. These findings for standalone treatments do not speak to practice recommendations and clinical applications for combined treatments, which should continue to be recommended and used according to the relevant literature and guidance [11, 20, 28, 86, 87].

Based on the included evidence, this review found that for CYP with anxiety, psychosocial and pharmacological treatments were equally effective at improving symptoms and increasing remission rates when used as standalone treatments; contrastingly, for CYP with depression, physical exercise interventions and IPT were the most effective standalone treatments, with promise from ACT, followed by CBT and other psychosocial therapies, then by antidepressants. Overall, psychosocial interventions and CBT were found to be more effective for treating anxiety than depression. Additional research in clinical settings may provide context for these findings which help adapt them to be applicable and feasible in everyday treatment of CYP, and to reflect the complexity of mental health conditions and interventions. In this rapidly developing research area, policymakers and practitioners should closely follow emerging evidence to ensure clinical practice reflects the most up-to-date evidence base. Recommendations to consider following this review include devaluing the use of MBIs in the treatment of anxiety, increasing consideration around the use of IPT and physical exercise interventions for depression, and closely following developments with IPT for anxiety and ACT for depression.

## Supplementary Information

Below is the link to the electronic supplementary material.

Supplementary file1 (DOCX 564 KB)

## Data Availability

A pre-print of the manuscript has been uploaded to OSF PsyArXiv for transparency and is openly available alongside the code and dataset: [(20)](https:/osf.io/preprints/psyarxiv/vaf8p_v1).
