# Supplementary material for: Effectiveness of interventions for anxiety and depression in children and young people: an umbrella review of meta-analyses
Source: Eur Child Adolesc Psychiatry. 2026 May 26;35(8):2445–76. doi: 10.1007/s00787-026-03064-w (PMC13652785; doi:10.1007/s00787-026-03064-w)
Supplement: Supplementary file 1 [file 787_2026_3064_MOESM1_ESM.docx]

**Effectiveness of interventions for anxiety and depression in children and young people:
an umbrella review of meta-analyses**

Isabella Morse^1*^ (ORCID 0009-0001-9910-4453),

Julia Mannes^1^ (ORCID 0000-0002-2236-5970),

Samantha Phippard^1^ (ORCID 0000-0003-3673-157X),

Fabienne dos Santos Sousa^1^ (ORCID 0009-0000-2643-0517),

Dihini Pilimatalawwe^1^ (ORCID 0009-0005-8819-6929)

Rani Samuel^2^,

Mohan Ganesalingam^1^,

Emma Rachel Rengasamy^1,3^ (ORCID 0000-0001-7059-9627),

Carmen Pinto^4^ (ORCID 0000-0003-3887-6289),

Matt Woolgar^5^ (ORCID 0000-0002-3618-0395),

Robbie Duschinsky^1^ (ORCID 0000-0003-2023-5328),

Barry Coughlan^1,6^ (ORCID 0000-0002-1484-6491)

1. Child Health and Development Group, Department of Public Health and Primary Care, University of Cambridge, Cambridge, United Kingdom
2. Lewisham CAMHS, South London and Maudsley NHS Foundation Trust, London, United Kingdom
3. Norfolk and Suffolk NHS Foundation Trust, Norwich, United Kingdom
4. South London and Maudsley NHS Foundations Trust, London, United Kingdom
5. Institute of Psychiatry, Psychology & Neuroscience (IoPPN), King’s College London, London, United Kingdom
6. Department of Social Sciences, National College of Ireland, Dublin, Ireland

*Corresponding Author Isabella Morse email: im524@cam.ac.uk

**Supplementary S1:** **Database search terms**

**Initial Searches**

**For PubMed**: 10 years ending on 20 August 2021;
Searchedpublished articles for…

(Child* OR Infant* OR Pediatric* OR Young OR Primary-age* OR Adolescen* OR Teen* OR Youth)

AND

(Anxiet* OR Depression OR "Internalizing difficulties" OR "Internalizing disorder*" OR "Emotional Distress")

AND

(Intervention* OR Treatment* OR Therap* OR Outreach OR Support OR Program*)

AND

(Metaanaly* OR Meta-analy* OR "quantitative review*" OR "quantitative synthesis" OR "quantitative review" OR meta-synthesis)

**For Medline, PsycINFO, Web of Science, Applied Social Sciences Index and Abstracts (ASSIA):** 5 years ending 1 Oct 2022;
**For PubMed:** from 20 August 2021 to 1 Oct 2022
Searched published articles for…

(Child* OR Infant* OR Pediatric* OR Young OR Primary-age* OR Adolescen* OR Teen* OR Youth)

AND

(Anxiet* OR Depression)

AND

(Intervention* OR Treatment* OR Therap* OR Outreach OR Support OR Program*)

AND

(Metaanaly* OR Meta-analy* OR "quantitative review*" OR "quantitative synthesis" OR "quantitative review" OR meta-synthesis)

**Updated Search**

**For PubMed:** from 1 Oct 2022 to 6 Feb 2026

(Child* OR Infant* OR Pediatric* OR Young OR Primary-age* OR Adolescen* OR Teen* OR Youth)

AND

(Anxiet* OR Depression)

AND

(Intervention* OR Treatment* OR Therap* OR Outreach OR Support OR Program*)

AND

(Metaanaly* OR Meta-analy* OR "quantitative review*" OR "quantitative synthesis" OR "quantitative review" OR meta-synthesis)

**Supplementary S2: Database Searches and Results Tables**

**Initial Search**

| # | Domain  [01.10.17- 01.10.22] |  | Search term | PubMed | Medline | PsycINFO | Web of Science | ASSIA |
| --- | --- | --- | --- | --- | --- | --- | --- | --- |
| 1 | Population |  | Child* |  |  |  |  |  |
|  |  |  | Infant* |  |  |  |  |  |
|  |  |  | Pediatric* |  |  |  |  |  |
|  |  |  | Young |  |  |  |  |  |
|  |  |  | Primary-age* |  |  |  |  |  |
|  |  |  | Adolescen* |  |  |  |  |  |
|  |  |  | Teen* |  |  |  |  |  |
|  |  |  | Youth |  |  |  |  |  |
|  |  | # | OR | 1,263,458 | 1,141,882 | 253,209 | 1,110,341 | 84,722 |
| 2 | Conditions |  | Anxiet* |  |  |  |  |  |
|  |  |  | Depression |  |  |  |  |  |
|  |  |  | "Internalizing difficulties" |  | *Not included in search* | *Not included in search* | *Not included in search* | *Not included in search* |
|  |  |  | "Internalizing disorder*" |  | *Not included in search* | *Not included in search* | *Not included in search* | *Not included in search* |
|  |  |  | "Emotional Distress" |  | *Not included in search* | *Not included in search* | *Not included in search* | *Not included in search* |
|  |  | # | OR | 198,337 | 198,402 | 115,737 | 273,783 | 36,755 |
| 3 | Interventions |  | Intervention* |  |  |  |  |  |
|  |  |  | Treatment* |  |  |  |  |  |
|  |  |  | Therap* |  |  |  |  |  |
|  |  |  | Outreach |  |  |  |  |  |
|  |  |  | Support |  |  |  |  |  |
|  |  |  | Program* |  |  |  |  |  |
|  |  | # | OR | 4,195,952 | 4,348,072 | 453,895 | 4,235,541 | 124,780 |
| 4 | Study Type |  | Metaanaly* |  |  |  |  |  |
|  |  |  | Meta-analy* |  |  |  |  |  |
|  |  |  | “quantitative review*” |  |  |  |  |  |
|  |  |  | “quantitative synthesis” |  |  |  |  |  |
|  |  |  | “quantitative review” |  |  |  |  |  |
|  |  |  | meta-synthesis |  |  |  |  |  |
|  |  | # | OR | 141,836 | 90,097 | 19,494 | 237,643 | 19,422 |
|  | Combined | #1 AND #2 AND #3 AND #4 | | 2,086    ***PubMed search completed including original search terms | 1,553 | 966 | 3,329 | 769 |

Note: Preliminary search was conducted 20 August 2021 in all five databases with a ten-year timeframe (from 20 August 2011) with the expanded search terms listed. This search produced 14,053 results. After reviewing the PubMed articles for study eligibility to establish a reasonable scale for the review, the volume of articles eligible for inclusion and the potential for significant study overlap led us to narrow the focus of the review to only anxiety and depression and to adapt to an updated five-year timeframe, and thus the search parameters were adjusted for the remaining databases accordingly. An updated search was run in PubMed to extract articles from 20 August 2021 to 1 October 2022, and all articles were rescreened with the updated eligibility criteria.

**Updated Search**

| # | Domain  [01.10.22 - 06.02.26] |  | Search term | PubMed |
| --- | --- | --- | --- | --- |
| 1 | Population |  | Child* |  |
|  |  |  | Infant* |  |
|  |  |  | Pediatric* |  |
|  |  |  | Young |  |
|  |  |  | Primary-age* |  |
|  |  |  | Adolescen* |  |
|  |  |  | Teen* |  |
|  |  |  | Youth |  |
|  |  | # | OR | 954,973 |
| 2 | Conditions |  | Anxiet* |  |
|  |  |  | Depression |  |
|  |  | # | OR | 170,565 |
| 3 | Interventions |  | Intervention* |  |
|  |  |  | Treatment* |  |
|  |  |  | Therap* |  |
|  |  |  | Outreach |  |
|  |  |  | Support |  |
|  |  |  | Program* |  |
|  |  | # | OR | 3,181,599 |
| 4 | Study Type |  | Metaanaly* |  |
|  |  |  | Meta-analy* |  |
|  |  |  | “quantitative synthesis” |  |
|  |  |  | “quantitative review” |  |
|  |  |  | meta-synthesis |  |
|  |  | # | OR | 139,647 |
|  | Combined | #1 AND #2 AND #3 AND #4 | | 2,087 |

Note: An updated search was conducted on 6 February 2026 in PubMed only to identify research published between the time of initial searching and publication of this umbrella review. Searching in PubMed only limited the potential for overlapping meta-analyses and ensured a rapid update was feasible.

**Supplementary S3: Inclusion and exclusion criteria by category**

| **Population** | **Study Design** | **Outcomes** |
| --- | --- | --- |
| Maximum participant age of 18 years | Systematic reviews  Meta-analyses | Focus on symptom improvement, remission, or other well-being/functioning outcomes |
| Symptoms of anxiety/ anxiety disorders present AND/OR depression/ depressive disorders as the primary consideration | Evaluations of active treatment interventions  Psychosocial, pharmacological, physical, or combination | At least four studies included per outcome/intervention |
| **NO** distinguishing group circumstances (i.e. natural disaster or trauma survivors, children in detention centers, children with chronic/fatal illnesses, other primary diagnosis group) | **NO** protocols for systematic reviews or other studies, single studies, umbrella reviews, book chapters | **NO** studies focusing on understanding levels of risk, prevalence, or association with outside factors; or studies focused on prevention in children without existing symptoms |

**Supplementary S4: List of data extracted from meta-analyses at full text review stage by category of data**

| **General Information** | **Demographic Information** | **Diagnoses & Intervention Information** | **Results** |
| --- | --- | --- | --- |
| Title | Number of participants | General Outcome (Anxiety or Depression) | Effect size of intervention outcomes |
| Year of Publication | Number of included studies/ comparisons | Specific Outcome (Diagnosis, symptoms, etc.) | Analysis method (random or fixed effects) |
| Author Names | Highest number of participants in a study | Outcome focus (Symptom severity, remission, etc.) | P-value of effect size |
|  | Lowest number of participants in a study | Sample context (recruited from clinical population or community population) | Standard error of effect size |
|  | Number of participants in intervention group | General Intervention Type (Psychosocial, Pharmacological, Physical) | Confidence interval of effect size |
|  | Number of participants in control group | Details of Intervention | Homogeneity score |
|  | Number of participants who are female | Follow-up measurements |  |
|  | Number of participants who are male | Mean number of treatment sessions |  |
|  | Number of participants who are another gender | Session duration (in range of minutes) |  |
|  | Number of participants who are white | Session duration (in range of weeks) |  |
|  | Number of participants who are non-white | Outcome measure (Self-report, parent-report, clinician-report, etc.) |  |
|  | Mean age of participants | Comparison group (treatment-control or pre-post intervention) |  |
|  | Median age of participants |  |  |
|  | Age range of participants |  |  |

# **Supplementary S5: R Coding packages and citations**

All analyses were conducted in R version 4.2.1 using the following packages: car,^1^ dplyr,^2^ ggplot2,^3^ ggsci,^4^ gt,^5^ kableExtra,^6^ knitr,^7^ metafor,^8^ patchwork,^9^ readxl,^10^ rstatix,^11^ scales,^12^ sjPlot,^13^ tidyr,^14^ tidyverse,^15^ writexl.^16^

See OSF PsyArXiv for full code and data: <https://osf.io/preprints/psyarxiv/vaf8p_v1>.

Package References

1.	Fox J, Weisberg S. An R Companion to Applied Regression [Internet]. Third. Thousand Oaks CA: Sage; 2019. Available from: https://socialsciences.mcmaster.ca/jfox/Books/Companion/

2.	Wickham H, François R, Henry L, Müller K, Vaughan D. dplyr: A Grammar of Data Manipulation [Internet]. 2023. Available from: https://CRAN.R-project.org/package=dplyr

3.	Wickham H. ggplot2: Elegant Graphics for Data Analysis [Internet]. Springer-Verlag New York; 2016. Available from: https://ggplot2.tidyverse.org

4.	Xiao N. ggsci: Scientific Journal and Sci-Fi Themed Color Palettes for “ggplot2” [Internet]. 2024. Available from: https://CRAN.R-project.org/package=ggsci

5.	Iannone R, Cheng J, Schloerke B, Hughes E, Lauer A, Seo J. gt: Easily Create Presentation-Ready Display Tables [Internet]. 2023. Available from: https://CRAN.R-project.org/package=gt

6.	Zhu H. kableExtra: Construct Complex Table with “kable” and Pipe Syntax [Internet]. 2024. Available from: https://CRAN.R-project.org/package=kableExtra

7.	Xie Y. knitr: A Comprehensive Tool for Reproducible Research in R. In: Stodden V, Leisch F, Peng RD, editors. Implementing Reproducible Computational Research [Internet]. Chapman and Hall/CRC; 2014. Available from: http://www.crcpress.com/product/isbn/9781466561595

8.	Viechtbauer W. Conducting meta-analyses in R with the metafor package. J Stat Softw. 2010;36(3):1–48.

9.	Pedersen TL. patchwork: The Composer of Plots [Internet]. 2025. Available from: https://CRAN.R-project.org/package=patchwork

10.	Wickham H, Bryan J. readxl: Read Excel Files [Internet]. 2023. Available from: https://CRAN.R-project.org/package=readxl

11.	Kassambara A. rstatix: Pipe-Friendly Framework for Basic Statistical Tests [Internet]. 2023. Available from: https://CRAN.R-project.org/package=rstatix

12.	Wickham H, Pedersen TL, Seidel D. scales: Scale Functions for Visualization [Internet]. 2023. Available from: https://CRAN.R-project.org/package=scales

13.	Lüdecke D. sjPlot: Data Visualization for Statistics in Social Science [Internet]. 2024. Available from: https://CRAN.R-project.org/package=sjPlot

14.	Wickham H, Vaughan D, Girlich M. tidyr: Tidy Messy Data [Internet]. 2023. Available from: https://CRAN.R-project.org/package=tidyr

15.	Wickham H, Averick M, Bryan J, Chang W, McGowan LD, François R, et al. Welcome to the tidyverse. J Open Source Softw. 2019;4(43):1686.

16.	Ooms J. writexl: Export Data Frames to Excel “xlsx” Format [Internet]. 2023. Available from: https://CRAN.R-project.org/package=writexl

**Supplementary S6: Quality of individual meta-analysis assessed with AMSTAR-2**

| **Study** | **Q1** | **Q2** | **Q3** | **Q4** | **Q5** | **Q6** | **Q7** | **Q8** | **Q9a** | **Q9b** | **Q10** | **Q11a** | **Q11b** | **Q12** | **Q13** | **Q14** | **Q15** | **Q16** | **Homogeneity** |
| --- | --- | --- | --- | --- | --- | --- | --- | --- | --- | --- | --- | --- | --- | --- | --- | --- | --- | --- | --- |
| *****Alemdar & Karaca | Yes | Partial Yes | No | Partial Yes | Yes | Yes | Yes | No | Yes | Yes | No | Yes | No | Yes | Yes | Yes | Yes | Yes | No |
| *****Alozkan-Sever et al | Yes | Yes | No | Partial Yes | Yes | Yes | Yes | Yes | Yes | N/A | No | Yes | N/A | Yes | Yes | Yes | Yes | Yes | No |
| Arnardóttir & Skarphedinsson | Yes | Yes | Yes | Yes | Yes | Yes | Yes | Partial Yes | Yes | N/A | No | Yes | N/A | Yes | Yes | Yes | Yes | Yes | N/A |
| Axelsdottir et al | Yes | Yes | No | Yes | Yes | Yes | Yes | Yes | Yes | N/A | No | Yes | N/A | Yes | Yes | Yes | Yes | Yes | No |
| Baker et al | Yes | Yes | No | Partial Yes | Yes | Yes | Yes | Partial Yes | Yes | N/A | No | Yes | N/A | Yes | Yes | Yes | Yes | Yes | No |
| Baourda et al | Yes | No | No | Partial Yes | Yes | Yes | Yes | Partial Yes | Yes | N/A | No | Yes | N/A | Yes | Yes | Yes | Yes | Yes | No |
| Campisi et al | Yes | Yes | No | Yes | Yes | Yes | Yes | Partial Yes | Yes | Yes | No | Yes | Yes | No | No | Yes | Yes | Yes | Yes |
| *****Carneiro et al | Yes | Yes | No | Partial Yes | Yes | Yes | Yes | Yes | Yes | N/A | No | Yes | N/A | No | No | Yes | Yes | Yes | No |
| Carnes et al (excluded from meta-analysis) | Yes | No | No | Partial Yes | No | No | Yes | Partial Yes | Yes | N/A | No | Yes | N/A | Yes | Yes | Yes | Yes | Yes | No |
| Cervin & Lundgren | Yes | No | No | Partial Yes | Yes | Yes | Yes | Partial Yes | Yes | N/A | No | Yes | N/A | No | No | Yes | Yes | Yes | Yes |
| *****Csirmaz et al | Yes | Partial Yes | No | Yes | Yes | Yes | Yes | Yes | Yes | N/A | No | Yes | N/A | Yes | No | Yes | Yes | Yes | Yes |
| Cuijpers et al (a) | Yes | Yes | No | Partial Yes | Yes | Yes | Yes | Partial Yes | Yes | N/A | No | Yes | N/A | Yes | Yes | Yes | Yes | Yes | No |
| *****Cuijpers et al (b) | Yes | Yes | No | Yes | Yes | Yes | Yes | Yes | Yes | N/A | No | Yes | N/A | Yes | Yes | Yes | Yes | Yes | No |
| Dippel et al | Yes | No | No | Partial Yes | Yes | Yes | Yes | Yes | Yes | N/A | No | Yes | N/A | Yes | Yes | Yes | Yes | Yes | No |
| Dobson et al | Yes | No | No | Partial Yes | No | No | Yes | Yes | Yes | N/A | Yes | Yes | N/A | Yes | Yes | Yes | Yes | Yes | Yes |
| Eckshtain et al (a) | Yes | No | No | Partial Yes | No | Yes | Yes | Yes | Partial Yes | N/A | No | Yes | N/A | Yes | Yes | Yes | Yes | No | No |
| Eckshtain et al (b) | Yes | No | No | Partial Yes | No | Yes | Yes | Yes | Partial Yes | N/A | Yes | No | N/A | Yes | Yes | Yes | Yes | Yes | No |
| Evans et al (excluded from meta-analysis) | Yes | Yes | No | Partial Yes | No | Yes | Yes | Partial Yes | Yes | N/A | No | Yes | N/A | Yes | Yes | Yes | Yes | Yes | Yes |
| Fulambarkar et al | Yes | No | No | Partial Yes | Yes | Yes | Yes | Yes | Yes | N/A | No | No | N/A | Yes | Yes | No | No | Yes | No |
| Guo et al (excluded from meta-analysis) | Yes | Partial Yes | No | Yes | Yes | Yes | Yes | Partial Yes | Yes | N/A | No | Yes | N/A | Yes | No | Yes | Yes | Yes | Yes |
| Hang et al | Yes | No | No | Partial Yes | Yes | Yes | Yes | Yes | Yes | N/A | No | Yes | N/A | Yes | Yes | Yes | Yes | Yes | Yes |
| Hetrick et al | Yes | Yes | Yes | Yes | Yes | Yes | Yes | Yes | Yes | N/A | Yes | Yes | N/A | Yes | Yes | Yes | Yes | Yes | N/A |
| Higinbotham et al | Yes | No | No | Partial Yes | Yes | Yes | Yes | Partial Yes | No | N/A | No | Yes | N/A | No | No | Yes | Yes | Yes | No |
| Hugh-Jones et al | Yes | Yes | No | Partial Yes | Yes | Yes | Yes | Yes | Yes | N/A | No | Yes | N/A | Yes | Yes | Yes | Yes | Yes | No |
| James et al | Yes | No | No | Yes | Yes | Yes | Yes | Yes | Yes | N/A | No | Yes | N/A | Yes | Yes | Yes | Yes | Yes | No |
| Jewell et al | Yes | Yes | Yes | Partial Yes | No | No | Yes | Yes | Yes | Yes | No | Yes | No | No | Yes | Yes | Yes | Yes | No |
| Keles & Idsoe | Yes | Yes | Yes | Partial Yes | Yes | Yes | Yes | Partial Yes | No | N/A | No | Yes | N/A | Yes | Yes | Yes | Yes | No | Yes |
| Krebs et al | Yes | No | No | Partial Yes | Yes | No | Yes | Partial Yes | Yes | N/A | No | Yes | N/A | Yes | Yes | Yes | Yes | Yes | Yes |
| Kreuze et al | Yes | No | No | Partial Yes | Yes | Yes | Yes | Yes | Yes | N/A | No | Yes | N/A | Yes | No | Yes | Yes | Yes | Yes |
| *****Li et al (a) | Yes | Yes | No | Yes | Yes | Yes | Yes | Yes | Yes | N/A | No | Yes | N/A | Yes | No | Yes | Yes | Yes | No |
| *****Li et al (b) | Yes | Yes | No | Yes | Yes | Yes | Yes | Yes | Yes | N/A | No | Yes | N/A | Yes | Yes | Yes | Yes | Yes | No |
| *****Liu et al | Yes | Yes | No | Partial Yes | Yes | Yes | Yes | Yes | Yes | N/A | No | No | N/A | Yes | Yes | Yes | Yes | No | No |
| *****López-Pinar et al | Yes | Yes | No | No | Yes | Yes | Yes | Yes | Yes | N/A | No | Yes | N/A | No | Yes | Yes | Yes | Yes | No |
| Luo & McAloon | Yes | Yes | Yes | Partial Yes | No | No | Yes | Partial Yes | Yes | N/A | No | Yes | N/A | Yes | Yes | Yes | Yes | Yes | Yes |
| Oberste et al | Yes | No | No | Partial Yes | Yes | Yes | Yes | Yes | Yes | Yes | No | Yes | N/A | Yes | Yes | Yes | Yes | Yes | Yes |
| Odgers et al | Yes | Yes | Yes | Partial Yes | No | No | Yes | Yes | Yes | N/A | No | Yes | N/A | Yes | Yes | Yes | Yes | Yes | No |
| Phillips & Mychailyszyn (excluded from meta-analysis) | No | No | Yes | Partial Yes | Yes | No | Yes | Partial Yes | No | No | No | Yes | No | No | No | No | Yes | Yes | No |
| Pu et al | Yes | No | No | Partial Yes | Yes | Yes | Yes | Partial Yes | Yes | N/A | No | Yes | N/A | Yes | Yes | Yes | Yes | Yes | Yes |
| Reyad et al | Yes | No | No | Partial Yes | No | No | No | Partial Yes | Yes | N/A | No | Yes | N/A | No | No | Yes | No | Yes | No |
| Schwartz et al | Yes | Yes | No | Partial Yes | Yes | Yes | No | Yes | Yes | N/A | No | Yes | N/A | Yes | Yes | Yes | Yes | Yes | Yes |
| Steains et al | Yes | No | Yes | Partial Yes | Yes | Yes | Yes | Partial Yes | Partial Yes | N/A | No | Yes | N/A | No | Yes | Yes | Yes | Yes | Yes |
| Stefánsdóttir et al | Yes | No | No | Partial Yes | Yes | Yes | Yes | Yes | Yes | N/A | Yes | Yes | N/A | Yes | Yes | Yes | Yes | Yes | No |
| Strawn et al | Yes | No | No | Partial Yes | No | No | Yes | Partial Yes | Yes | N/A | Yes | Yes | N/A | Yes | Yes | Yes | No | Yes | N/A |
| Tejada-Gallardo et al | Yes | Yes | Yes | Partial Yes | Yes | Yes | Yes | Yes | Yes | Yes | No | Yes | No | Yes | Yes | Yes | Yes | Yes | Yes |
| Teng et al | Yes | No | No | Yes | Yes | Yes | Yes | Partial Yes | Yes | N/A | Yes | Yes | N/A | Yes | Yes | Yes | Yes | Yes | Yes |
| *****Tindall et al | Yes | Partial Yes | No | Yes | Yes | Yes | Yes | Yes | Yes | N/A | No | Yes | N/A | No | Yes | Yes | Yes | Yes | Yes |
| *****van Aswegen et al | Yes | Partial Yes | No | Yes | Yes | Yes | Yes | Yes | Yes | N/A | No | Yes | N/A | Yes | Yes | Yes | Yes | No | No |
| Wang et al | Yes | Yes | No | Partial Yes | Yes | Yes | Yes | Yes | Yes | N/A | No | Yes | N/A | Yes | Yes | Yes | Yes | Yes | No |
| Waraan et al (excluded from meta-analysis) | Yes | Yes | No | Yes | Yes | Yes | Partial Yes | Yes | Yes | N/A | No | Yes | N/A | Yes | Yes | Yes | Yes | Yes | No |
| Whiteside et al | Yes | Yes | No | Partial Yes | Yes | Yes | No | Partial Yes | Yes | N/A | No | Yes | N/A | Yes | Yes | No | No | Yes | N/A |
| Yang et al (a) | Yes | No | Yes | Partial Yes | Yes | Yes | Yes | Partial Yes | Yes | N/A | No | Yes | N/A | Yes | Yes | Yes | Yes | Yes | Yes |
| Yang et al (b) | Yes | No | No | Partial Yes | Yes | Yes | Yes | Partial Yes | Yes | N/A | No | Yes | N/A | No | Yes | Yes | Yes | Yes | Yes |
| *****Yang et al (c) | Yes | No | No | Partial Yes | Yes | Yes | Yes | Partial Yes | Yes | N/A | No | Yes | N/A | Yes | No | Yes | Yes | Yes | No |
| *****Zhang et al (a) | Yes | Partial Yes | No | Partial Yes | Yes | Yes | Yes | Yes | Yes | N/A | No | Yes | N/A | No | No | Yes | Yes | Yes | No |
| *****Zhang et al (b) | Yes | No | Yes | Partial Yes | Yes | Yes | Yes | Yes | Yes | N/A | No | Yes | N/A | No | No | Yes | Yes | Yes | Yes |
| Zhou et al (a) | Yes | Partial Yes | No | Partial Yes | Yes | Yes | Yes | Yes | Yes | N/A | No | Yes | N/A | Yes | Yes | Yes | Yes | Yes | N/A |
| *****Zhou et al (b) | Yes | Yes | No | Partial Yes | Yes | Yes | Yes | Yes | Yes | N/A | No | Yes | N/A | No | Yes | Yes | Yes | Yes | No |

Note: AMSTAR-2 scores across individual meta-analysis. 
Q= Question. See https://amstar.ca/docs/AMSTAR-2.pdf for a list of the AMSTAR 2 questions.
AMSTAR-2: A MeaSurement Tool to Assess systematic Reviews version 2.
*****Studies from updated search

**Supplementary S7: Effect size distribution plots for anxiety and depression**

**ES Facet Plot**

**General ES Distribution Plot**

**Supplementary S8: Potential moderator data: percentages of missing data from all effect sizes**

## **Percent of missing moderator data from all effect sizes: frequency and duration**

| **Potential Moderator** | Number of Sessions - mean | Session duration - min | Treatment Duration - range in weeks |
| --- | --- | --- | --- |
| **Percent Available Data** | 32.7% | 12.2% | 41.6% |
| **Percent Missing Data** | 67.3% | 87.8% | 58.4% |

## **Percent of missing moderator data from all effect sizes: population characteristics**

| **Potential Moderator** | Gender | Ethnicity | Age - mean | Age - median | Age - range |
| --- | --- | --- | --- | --- | --- |
| **Percent Available Data** | 9.6% | 1.8% | 6.6% | 0% | 40.6% |
| **Percent Missing Data** | 90.4% | 98.2% | 93.4% | 100% | 59.4% |
